# Adaptive evolution of stress response genes in parasites aligns with host niche diversity

**DOI:** 10.1186/s12915-024-02091-w

**Published:** 2025-01-13

**Authors:** Armando J. Cruz-Laufer, Maarten P. M. Vanhove, Lutz Bachmann, Maxwell Barson, Hassan Bassirou, Arnold R. Bitja Nyom, Mare Geraerts, Christoph Hahn, Tine Huyse, Gyrhaiss Kapepula Kasembele, Samuel Njom, Philipp Resl, Karen Smeets, Nikol Kmentová

**Affiliations:** 1 Faculty of Sciences, Centre for Environmental Sciences, Research Group Zoology: Biodiversity and Toxicology, UHasselt – Hasselt University, Diepenbeek, Belgium; 2 Systems Ecology and Resource Management Research Unit (SERM), Université Libre de Bruxelles-ULB, Brussels, Belgium; 3 Natural History Museum, University of Oslo, Oslo, Norway; 4 Department of Biological Sciences, University of Botswana, Gaborone, Botswana; 5 Department of Biological Sciences, University of Ngaoundéré, Ngaoundéré, Cameroon; 6 Department of Management of Fisheries and Aquatic Ecosystems, Institute of Fisheries, University of Douala, Douala, Cameroon; 7 Department of Biology, Evolutionary Ecology Group – EVECO, University of Antwerp, Antwerp, Belgium; 8 Institute of Biology, University of Graz, Graz, Austria; 9 Department of Biology, Royal Museum for Central Africa, Tervuren, Belgium; 10 Unité de Recherche en Biodiversité Et Exploitation Durable Des Zones Humides (BEZHU), Faculté Des Sciences Agronomiques, Université de Lubumbashi, Lubumbashi, Democratic Republic of the Congo; 11 Aquatic and Terrestrial Ecology, Operational Directorate Natural Environment, Royal Belgian Institute for Natural Sciences, Brussels, Belgium

**Keywords:** Comparative genomics, Positive selection, Monopisthocotyla, Heat shock proteins, Oxidative stress

## Abstract

**Background:**

Stress responses are key the survival of parasites and, consequently, also the evolutionary success of these organisms. Despite this importance, our understanding of the evolution of molecular pathways dealing with environmental stressors in parasitic animals remains limited. Here, we tested the link between adaptive evolution of parasite stress response genes and their ecological diversity and species richness. We comparatively investigated antioxidant, heat shock, osmoregulatory, and behaviour-related genes (*foraging*) in two model parasitic flatworm lineages with contrasting ecological diversity, *Cichlidogyrus* and *Kapentagyrus* (Platyhelminthes: Monopisthocotyla), through whole-genome sequencing of 11 species followed by in silico exon bait capture as well as phylogenetic and codon analyses.

**Results:**

We assembled the sequences of 48 stress-related genes and report the first *foraging* (*For*) gene orthologs in flatworms. We found duplications of heat shock (*Hsp*) and oxidative stress genes in *Cichlidogyrus* compared to *Kapentagyrus*. We also observed positive selection patterns in genes related to mitochondrial protein import (*Hsp*) and behaviour (*For*) in species of *Cichlidogyrus* infecting East African cichlids—a host lineage under adaptive radiation. These patterns are consistent with a potential adaptation linked to a co-radiation of these parasites and their hosts. Additionally, the absence of cytochrome P450 and kappa and sigma-class glutathione S-transferases in monogenean flatworms is reported, genes considered essential for metazoan life.

**Conclusions:**

This study potentially identifies the first molecular function linked to a flatworm radiation. Furthermore, the observed gene duplications and positive selection indicate the potentially important role of stress responses for the ecological adaptation of parasite species.

**Supplementary Information:**

The online version contains supplementary material available at 10.1186/s12915-024-02091-w.

## Background

Evolutionary theory predicts that a species’ ability to maintain homeostasis against environmental stressors fundamentally affects its adaptive potential [1]. This paradigm also applies to metazoan parasites. Parasites cause many neglected tropical diseases in humans [2] but remain often overlooked as groups of pathogens, which also applies to research on their stress responses. Stress responses might be of high relevance to parasite evolution due to their role in parasite adaptation. Stress responses can determine a parasite's infectivity and virulence [3, 4]. Effective stress responses can also increase fitness of individuals and populations (microevolution) and permit species to expand host repertoires and geographical ranges, which may give rise to new parasite species and diseases (macroevolution) [5]. Furthermore, understanding parasite adaptation matters in a world where human activity promotes the rise of emerging infectious diseases as environmental disturbance creates new ecological opportunities for parasite species [6, 7]. Nonetheless, research on parasite stress responses remains largely limited to few well-known human-infecting species for the purpose of drug development [8, 9]—or studies focus on macroevolutionary adaptations of major parasite clades, e.g. flatworm classes or insect orders [10, 11]. Stress response pathways are rarely comparatively analysed below the level of these major lineages. Here, we aim to address this knowledge gap on how stress response systems evolve in parasite lineages that are closely related and functionally alike.

In parasitology, the ability to use a broad spectrum of resources, i.e. host species, is often considered indicative of an increased adaptive potential, specifically in ectoparasites, which are directly exposed to the environmental stressors experienced by their hosts. Several stress-related proteins have been characterised as determining host usage in parasites, e.g. in insects [12], nematodes [13], and fungi [14], including antioxidant enzymes dealing with reactive oxygen species (oxidative stress response), heat shock proteins assisting with protein folding, and aquaporins dealing with osmotic stress. Animals may also respond to environmental stressors through behavioural changes. The *foraging* (*For*) gene of *Drosophila melanogaster* Meigen, 1830 and other species are among the best-known examples of genes determining behavioural differences [15].

**Fig. 1:**
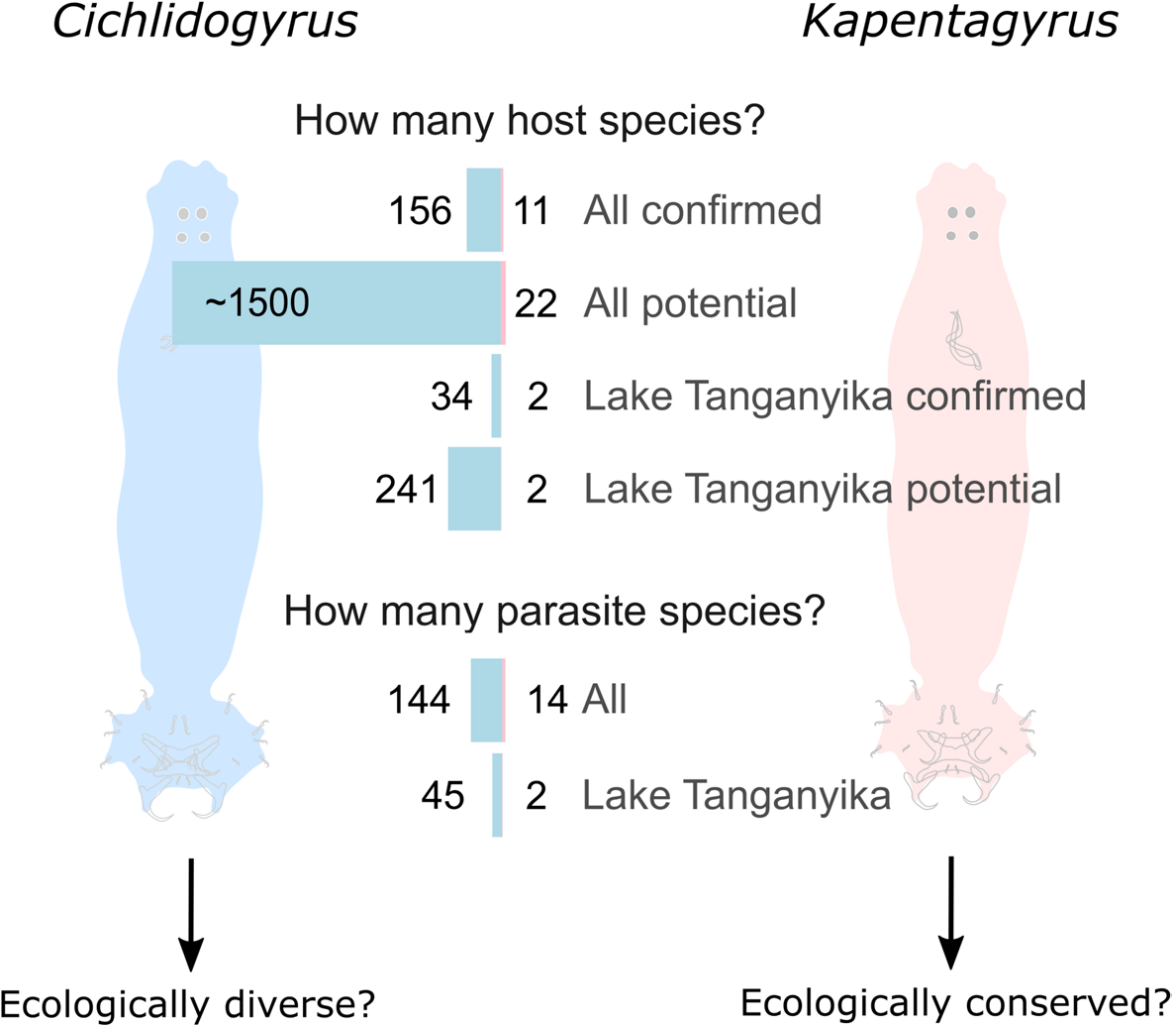
The two flatworm parasite lineages *Cichlidogyrus* and *Kapentagyrus* differ substantially in species richness and host diversity. Species of *Cichlidogyrus* infect the gills of the hyperdiverse African cichlid fishes that include the adaptive radiations of Lake Tanganyika in East Africa [118]. Species of *Kapentagyrus* infect the gills of African freshwater clupeid fishes, an ecologically conserved group of 22 species inhabiting only pelagic environments of lakes and rivers [89]. Based on these differences, we hypothesise that stress responses of *Cichlidogyrus* have adapted to this enhanced ecological diversity of their hosts

Monogenean flatworms (recently split into Monopisthocotyla Brabec, Salomaki, Kolísko, Scholz & Kuchta, 2023 and Polyopisthocotyla Brabec, Salomaki, Kolísko, Scholz & Kuchta, 2023) offer several advantages for comparatively addressing the evolution of stress response in parasites. They have single-host life cycles and the host preferences of various monogenean groups have been studied in detail, ranging from host specialists to generalists. Here, we focus on closely related lineages *Cichlidogyrus* Paperna, 1960 and *Kapentagyrus* Kmentová, Gelnar & Vanhove, 2018 [16]. Species of *Cichlidogyrus* and *Kapentagyrus* infect host lineages (African cichlid vs. freshwater clupeid fishes) with contrasting species richness and ecological diversity (Fig. 1). Species of *Cichlidogyrus* are parasites of cichlid fishes, the one of the most species-rich and ecological diverse group of fishes [17]. One subclade (*Cichlidogyrus* spp. infecting East African cichlids) is reported from a host lineage that has undergone multiple rapid diversification events (adaptive radiations) in its recent evolutionary history [18], coinciding with a high parasite species richness (Lake Tanganyika: *n* = 45, total: *n* = 144) (Fig. 1). Species of *Kapentagyrus* are parasites of African freshwater clupeids, all of which inhabit pelagic environments of rivers and lakes [19]. This niche conservatism is reflected in a much lower number of parasite species (*n* = 14). Here, we explore the diversity and adaptive evolution of genes encoding antioxidant enzymes, heat shock proteins, aquaporins, and *For* orthologs*.* We hypothesise duplication and positive selection in stress genes of *Cichlidogyrus* compared to *Kapentagyrus*, which is species-poor and infects a species-poor, ecologically conserved host lineage. With whole-genome sequencing data of 11 species, our study provides the largest genomic dataset from a single flatworm lineage to date. With 345 single-copy orthologs and 48 stress gene models, we present the most extensive multi-species analysis of stress genes in parasitic flatworms. Our study highlights the role of stress responses in the adaptive evolution of parasites.

## Results

### Species trees

**Fig. 2:**
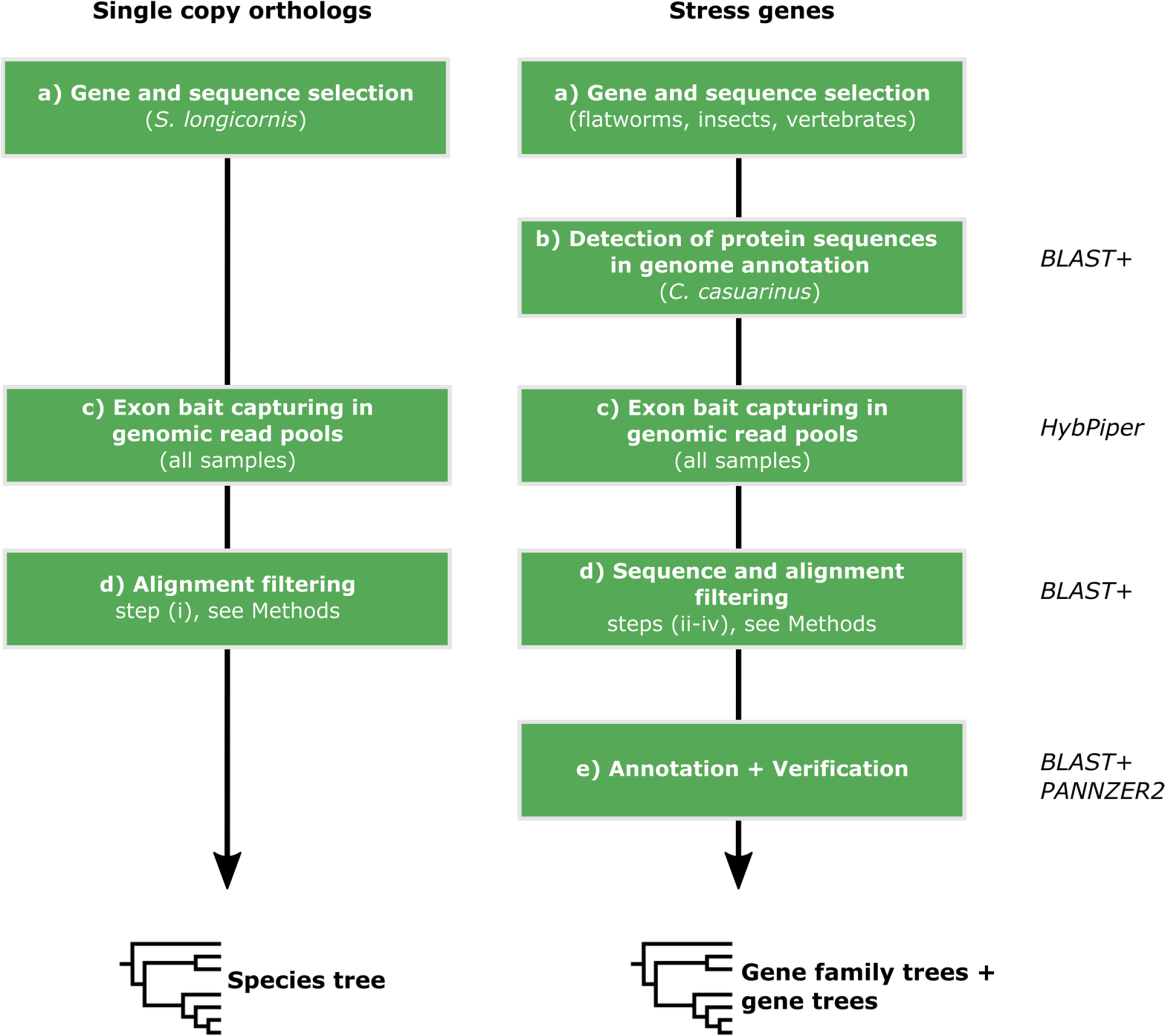
Schematic bioinformatic strategy for detecting single-copy orthologs (SCOs) and orthologs of stress genes in monogenean whole-genome short-reads. SCO sequences were used to infer the species tree and stress gene sequences for gene family trees and gene trees. **a** Bait sequences were chosen [*S. longicornis* was selected for SCOs; other organisms (non-monogenean flatworms, insects, vertebrates) were selected for stress genes due to lack of monogenean sequences]. **b** Orthologs of these sequences were detected in an annotated genome of *Cichlidogyrus casuarinus* (only stress genes). **c** The putative protein sequences of *S. longicornis* (SCOs)/*C. casuarinus* (stress genes) were used as baits for exon bait capture in the sequencing read pools of species of *Cichlidogyrus* and *Kapentagyrus* through *HybPiper* [99]. **d** Contaminant, variant, low–species coverage, and truncated sequences were filtered from the alignments. **e** Sequences were annotated through *PANNZER2* [103] (**e**)

**Fig. 3:**
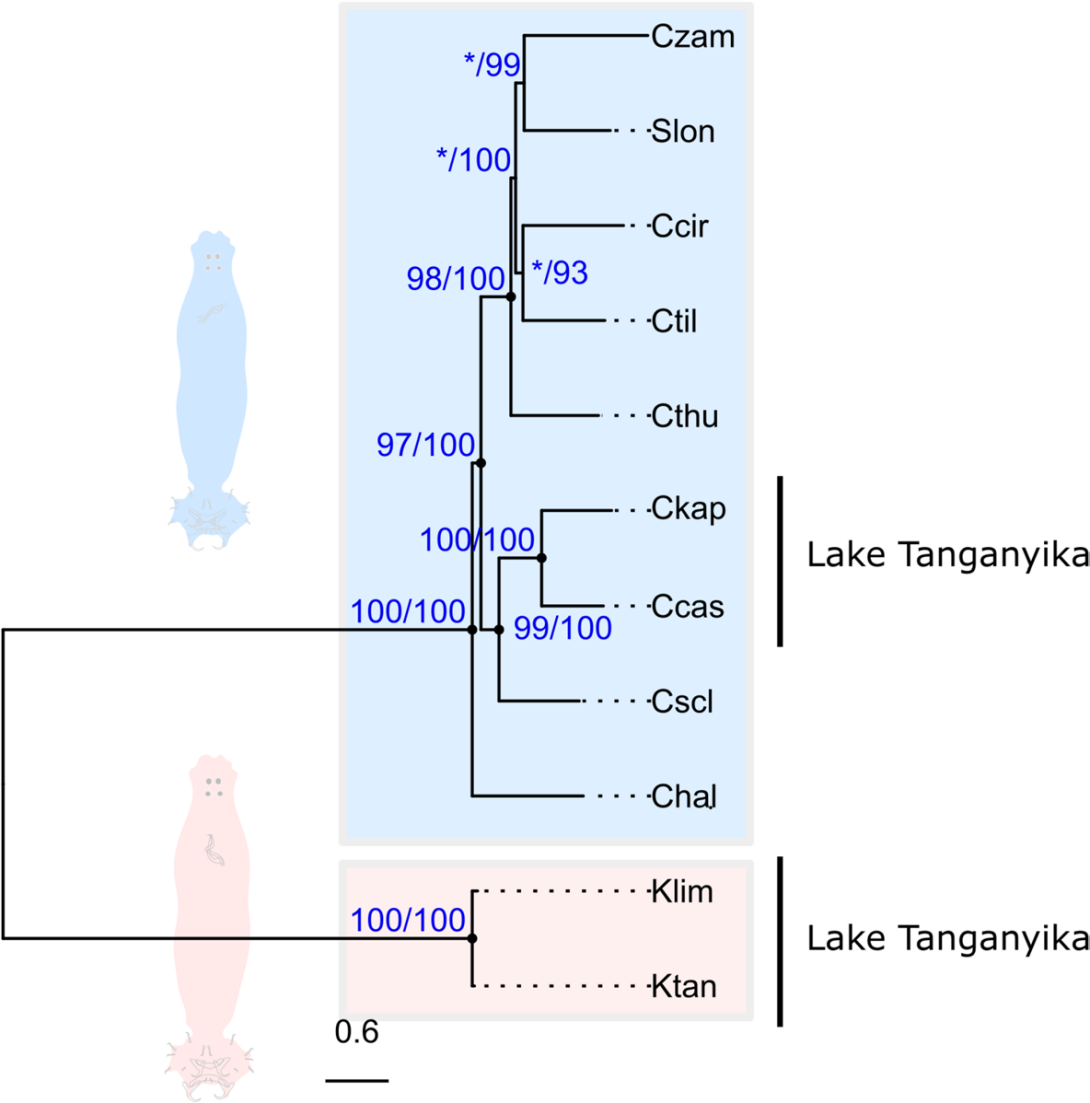
Species tree of *Cichlidogyrus* and *Kapentagyrus* inferred from 277 single-copy orthologs based on a subset of genes selected by Caña-Bozada et al. [20], who used the OMA pipeline. For the tree inferred from orthologs from the BUSCO pipeline, see Additional File 1. Support values: ultrafast bootstraps (UF-Boot)/Shimodaira-Hasegawa-like approximate likelihood ratio tests (SH-aLRT) (see Methods), asterisks (*) indicate support below threshold (UF-Boot ≤ 95, SH-aLRT ≤ 80). Abbreviations: Ccas–*Cichlidogyrus casuarinus*, Ccir–*C. cirratus*, Chal–*C. halli*, Ckap–*C.* sp. ‘kapembwa’, Cscl–*C. sclerosus*, Cthu–*C. thurstonae*, Ctil–*C. tilapiae*, Czam–*C. zambezensis*, Slon–*Scutogyrus longicornis*, Klim–*Kapentagyrus limnotrissae*, Ktan–*K. tanganicanus*, Lake Tanganyika–species endemic to Lake Tanganyika. Scale bar: estimated number of substitutions per site

As a phylogenetic backbone for downstream analyses, we inferred the evolutionary history of the two monogenean parasite lineages through phylogenomic analyses of single-copy ortholog genes. We assembled the nucleotide sequences of conserved single copy genes via in silico exon bait capture using orthologs of *Scutogyrus longicornis* (Paperna & Thurston, 1969) [20] as bait (Fig. 2a, c; single-copy orthologs). After alignment filtering (Fig. 2d), we retained 277 (OMA tree, Fig. 3) and 86 (BUSCO tree, Additional File 1) gene alignments. *Cichlidogyrus* and *Kapentagyrus* form well-supported monophyletic groups (Fig. 3). High support is also found for a clade of species of *Cichlidogyrus* from Lake Tanganyika in East Africa (Fig. 3, Clade Lake Tanganyika).

### Copy numbers and phylogenetic patterns of stress genes

**Fig. 4:**
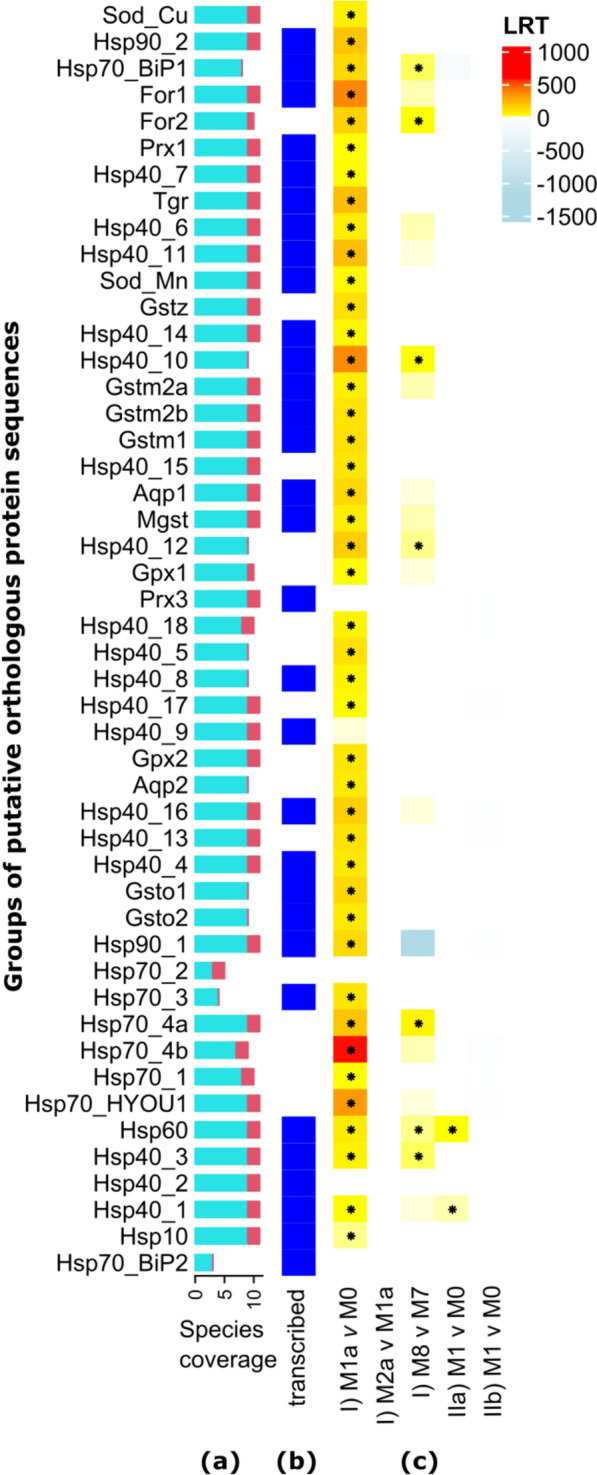
Detected putative stress gene orthologs (protein sequences) including species coverage (cyan = *Cichlidogyrus*, red = *Kapentagyrus*) (**a**), presence in transcriptome annotation (blue = present) (**b**), and hypothesis testing of different models for detecting positively selected gene sites (I, IIa, IIb) (**c**) with * indicating *P* < 0.05 for test results and the colour scale indicating the likelihood ratio test statistics (LRT) (see the ‘Methods’ section). Rows and columns of the GO heatmap are automatically sorted through Euclidean distances as implemented in *ComplexHeatmap*. For an extended version of this figure with GO term labels, see Additional File 6

Following preparation of the bait files using a genome annotation of *Cichlidogyrus casuarinus* Pariselle, Muterezi Bukinga & Vanhove, 2015 (see Additional File 2 [21–50]; Fig. 2a: single-copy orthologs) (WGS accession: JBJKFK000000000), search sequences of non-monogenean flatworms and other organisms (Fig. 2a: stress genes), and in-situ exon bait capture (Fig. 2c), we assembled nucleotide sequences of 48 putative stress genes of 11 monogenean species (Fig. 4a). The sequences of the 42 target genes included functional groups of their expected gene family (Fig. 4b, Additional File 3). A majority (63%) of the sequences matched with the reference transcriptome data of *S. longicornis* [51] (> 95% identity and query coverage) (Fig. 4c), but three out of nine heat shock *Hsp70* genes and all glutathione peroxidase (*Gpx*) and aquaporin (*Aqp*) variants were not found in the transcriptome. It falls outside the scope of the current study why these genes were not detected in the reference transcriptome, and it can only be speculated whether this is for biological (real absence) or methodological reasons (e.g. sequencing depth).

For most targeted stress genes, we found a single copy per sequencing read pool (Additional File 4). We detected deviations regarding copy numbers in the draft genome annotation (*C. casuarinus*) compared to the search sequences of other flatworm parasites, i.e. two *Gpx* (+ 1 vs.* Schistosoma mansoni*), four glutathione *Gstm* (-6 vs.* Echinococcus multilocularis*), two peroxiredoxin *Prx* (-1 vs.* S. mansoni*), two aquaporin *Aqp* genes (+ 1 vs.* S. mansoni*). No copies of cytochrome P450 genes (*Cyp*) and several glutathione families (*Gsta*, *Gsto*, *Gstp*, *Gsts*, and *Gstk*) were detected neither in *C. casuarinus* (Additional File 3) and data produced in this study, nor other published monogenean genomes (see the ‘Methods’ section) using *tblastn*. *Gpx*, *Gstm*, *Prx*, and all heat shock protein orthologs except *Hsp10* were flagged by *HybPiper* for potential paralogs (Additional File 4). The read pools in this study were generated from pooled individuals. To avoid counting allelic variants in the population as paralogs, highly similar sequences from the same species were excluded from downstream analyses using phylogenetic inference and manual curation. The filtered variants are listed in Additional File 3.

**Fig. 5:**
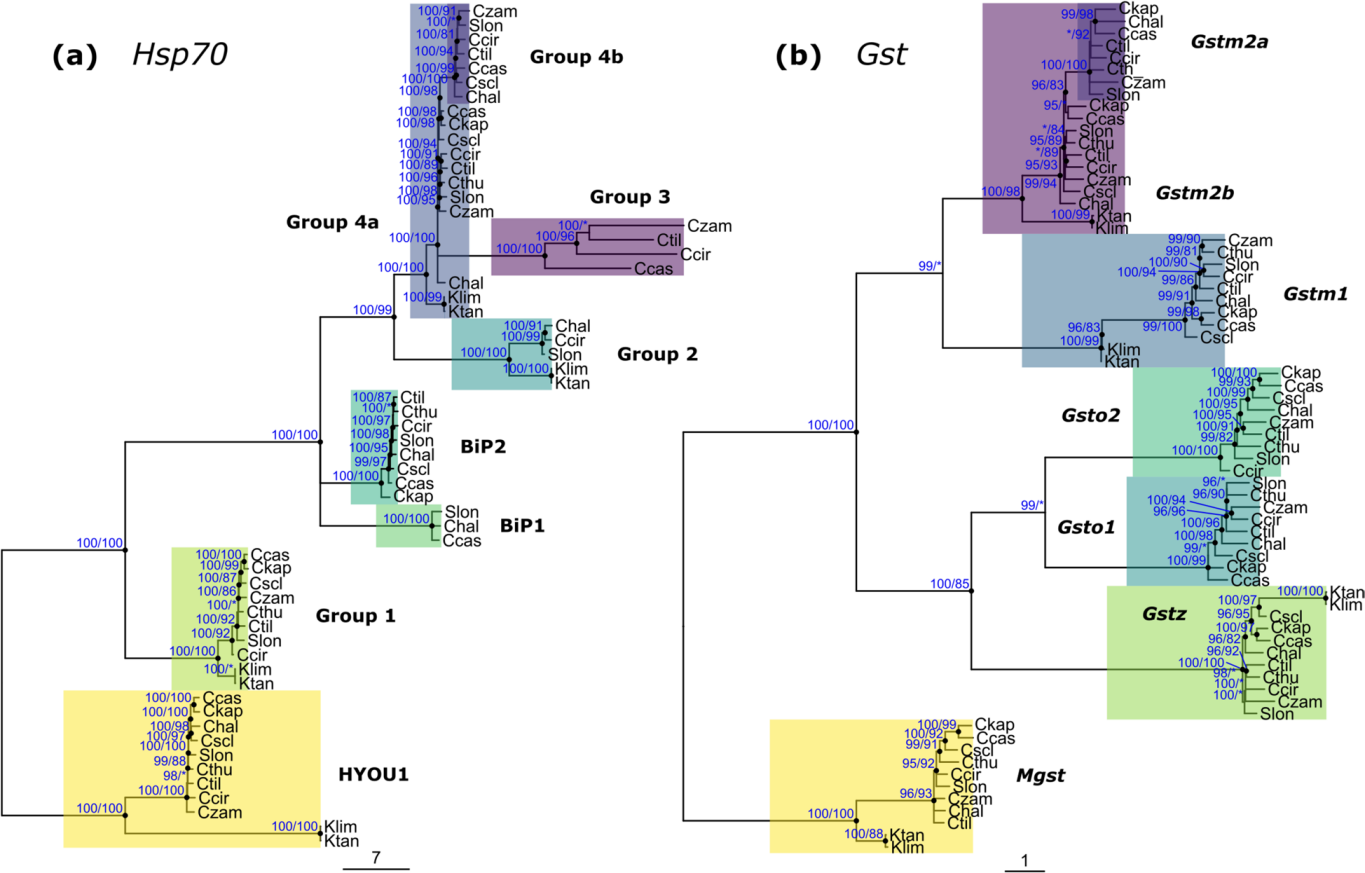
Maximum likelihood topologies of gene family trees and gene trees of species of *Cichlidogyrus* and *Kapentagyrus*. For abbreviation of species names, support values, and scale bars, see Fig. 3. **a** Gene models of the 70 kDa heat shock protein family (*Hsp70*). Group Hyou1 (hypoxia upregulated 1), BiP1 (endoplasmatic reticulum chaperone binding protein 1), and BiP2 refer to annotations assigned through *PANNZER2* (see Additional File 3); the remaining groups are numbered consecutively. **b** Gene models of the glutathione *S*-transferase (*Gst*) superfamily. Groups are named after *Gst* classes of the bait sequences and numbered consecutively. Group 4 *Hsp70* and Group *Gstm2* show potential duplication events (or gene losses) with two copies of the gene for species of *Cichlidogyrus* but only a single one for species of *Kapentagyrus*

For the *Hsp70* family, a multitude of paralogs were flagged for all but two bait sequences (Additional File 4). After filtering, we detected seven well-supported groups of *Hsp70* sequences in *Cichlidogyrus* (Fig. 5a), three of them not detected in *Kapentagyrus* (groups 3, 4a, and 4b). Through sequence similarity comparison with characterised *Hsp70* genes, three *Hsp70* groups were predicted to have highly specific functions: the hypoxia upregulated 1 (HYOU1) gene and the endoplasmatic reticulum chaperone binding proteins 1 (BIP1) and 2 (BIP2). Notably, group 4 constituted two orthologs for *Cichlidogyrus*, but only a single ortholog for *Kapentagyrus*. Group 3 appeared nested in group 4 (Fig. 5a), but this position might occur due to genetic saturation between the highly divergent *Hsp70* groups causing long-branch attraction (see [52]).

For the *Gst* families, we detected seven phylogenetic clusters (Fig. 5b). The mu-class (*Gstm*) sequences did not group according to the four bait sequences of *C. casuarinus*; hence, group names were reassigned according to the three *Gstm* clades inferred from the phylogenetic tree (Fig. 5b). Notably, *Gstm2* includes two copies for most species of *Cichlidogyrus* with identical gene ontology (GO) terms (Fig. 3) but only one for *Kapentagyrus* (Fig. 5b). We consider this absence of a copy as informative as the high sequence similarities of *Gstm2a* and *Gstm2b* (71–77% identical nucleotides) suggest that orthologs of *Gstm2a* in *Kapentagyrus* should have been detected if present. Other than the absence of *Gsto* in *Kapentagyrus* (mentioned above), no further copy number differences between the target species were detected in *Gst*.

**Fig. 6:**
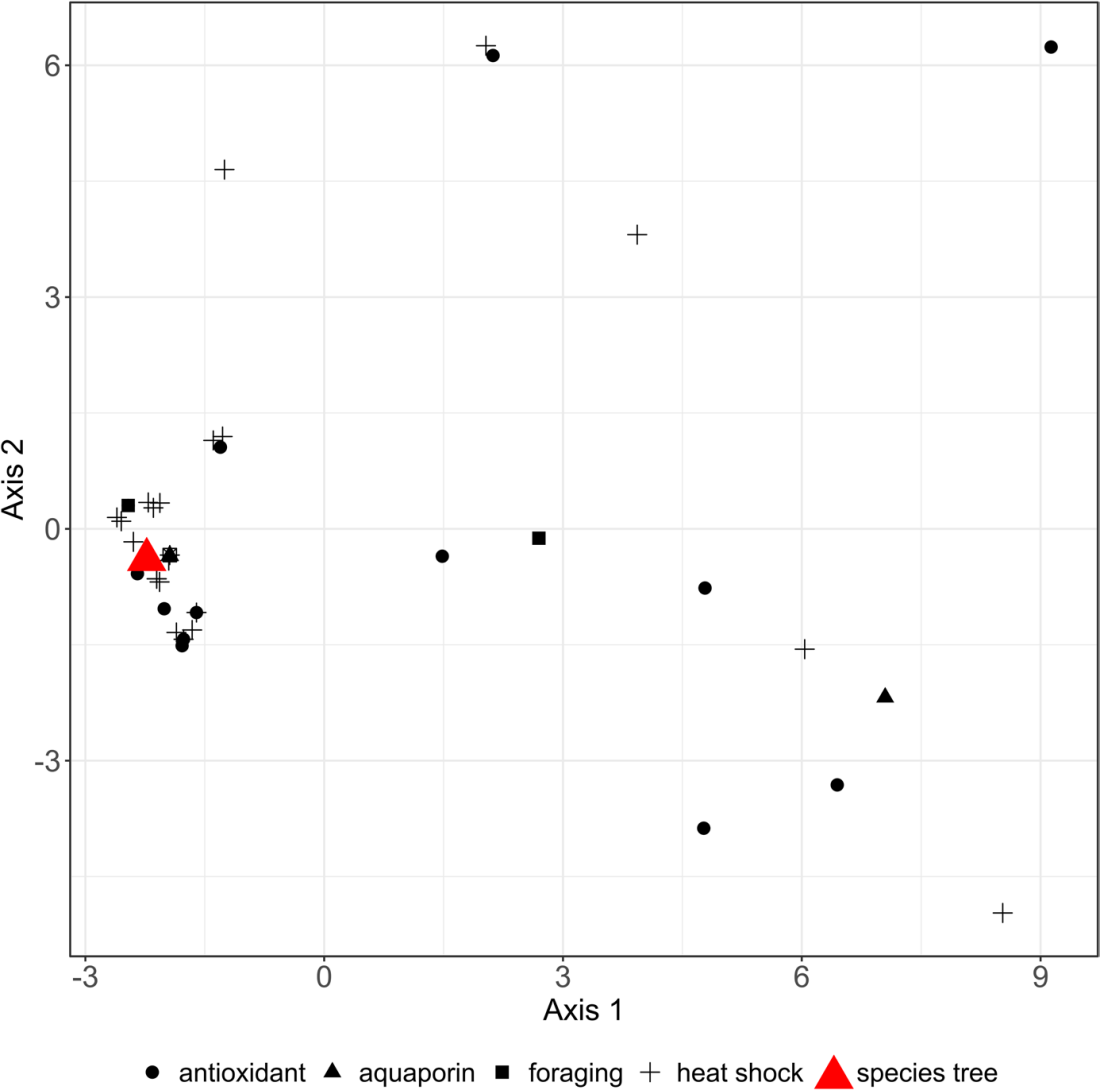
First two axes (49% of total variation) of multidimensional scaling analysis of gene trees of antioxidant enzymes, *for* orthologs, aquaporins, and heat shock proteins, with some gene tree topologies deviating from the two species trees (highlighted in red) but not forming clusters based on gene function or family

The species tree topologies (OMA vs. BUSCO orthologs) of *Cichlidogyrus* were highly similar to each other (Kendall-Colijn distance: 0) in a multidimensional scaling (MDS) visualisation when excluding species of *Kapentagyrus* (Fig. 6). For all assembled stress genes, gene trees involving the target species were constructed. Some stress gene trees (Additional File 5) deviated from the species tree topology (but unrelated to selection pressures, see below). This topological variation of stress gene tree topologies followed no apparent patterns based on gene function or family (Fig. 6).

### Detection of positive selection

Positive selection is the process by which gene variants that provide a fitness benefit dominate a population over time [53]. To investigate patterns of adaptive evolution in stress genes, we inferred positive selection from the ratio of substitution rates at nonsynonymous and synonymous sites in protein-coding sequences (d_N_/d_S_). We aimed to test whether the genes investigated here show signatures of positive selection regimes and whether differences are present between clades of *Cichlidogyrus* and *Kapentagyrus*. Our analyses revealed that seven stress genes had positively selected sites including one *for* and six *Hsp* genes (I, Fig. 4d). For clade-specific tests, we detected no differences between *Cichlidogyrus* and *Kapentagyrus* (IIa), but two *Hsp* genes had positively selected sites in East African species of *Cichlidogyrus* (IIb) (Fig. 4d), i.e. the mitochondrial molecular chaperone gene *Hsp60* as well as a putative *Hsp40* ortholog of the human DnaJ heat shock protein family (HSP40) member A1 (DNAJA1).

## Discussion

Stress responses are key factors influencing the ability of parasites to infect their hosts. The current understanding of ecological drivers of the evolution of parasite stress genes is limited as most studies only compare phylogenetically and ecologically distant species. In particular, the role of stress-related genes in adaptive evolution and speciation of metazoan parasites has never been comprehensively addressed.

We detected several unique stress response features in the targeted monogenean genomes. The absence of the cytochrome P450 gene family (*Cyp*) and glutathione *S*-transferase (*Gst*) sigma- (*Gsts*) and kappa-classes (*Gstk*) is remarkable. CYP enzymes are mainly involved in the (oxidative) metabolism of various endogenous and exogenous compounds and are conserved across almost the entire tree of life [54]. All GST members serve for cellular protection as detoxification enzymes, and *Gsts* and *Gstk* genes were previously reported from all flatworm genomes [55], except for those of monogenean species [11, 32, 56, 57]. The straightforward recovery and assembly of single-copy orthologs indicates the absence of *Cyp*, *Gsts*, and *Gstk* orthologs is real and not caused by low sequencing depth (see estimated coverages, Additional File 6 [58–60]). Although considered unlikely, highly divergent functional monogenean *Cyp*, *Gsts*, and *Gstk* may have remained undetected (see also a proposed peroxin gene in a parasitic protozoan [61]). The loss of *Cyp* has hitherto only been reported from protozoan parasites [62–64]. In other parasitic flatworms, i.e. flukes and tapeworms [55], only a single variant was reported that still fulfils vital functions [65]. The gene family has also been reduced in parasitic crustaceans, e.g. salmon lice have the lowest known *Cyp* copy number of any arthropod [66], which may reflect an evolutionary trend of *Cyp* contractions in metazoan parasites. For *Gst*s and *Gstk*, no losses have been reported in parasitic flatworms so far, but *Gsto* genes were reported absent in tapeworms [67]. Evolutionary loss of genes and gene functions has also repeatedly been observed elsewhere in parasites, e.g. peroxisomal functions in parasitic protozoans, flatworms, roundworms [68], and crustaceans [66]. These losses have in part been attributed to the r-selected traits of many parasites (e.g. high fecundity, few resources for individual offspring) suggesting that some gene losses might be related to stress response mechanisms [68]. *Cyp* and *Gst* gene family contractions and losses in monogeneans may fit this pattern.

The detection of several stress gene families in our target species is the first for monogenean flatworms. We noticed two gene copies of glutathione peroxidase (*Gpx*), two peroxiredoxins (*Prx*), six cytosolic glutathione *S-*transferases (*cGst*), and two aquaporins (*Aqp*) (Additional File 3). Monogeneans, thus, differ from other parasitic flatworms, with tapeworms and flukes presenting one *Gpx*, three *Prx*, 12 *cGst*, and one to three *Aqp* [55, 69] copies. As the functions of these antioxidant enzymes are the reduction of hydrogen peroxide to water (GPX, PRX) and alkyl hydroperoxides to alcohol (PRX), detoxification (cGST), and osmoregulation (AQP), gene family contractions/expansions could provide valuable insight into the functional evolution of parasitic flatworms. For instance, increased *Gpx* copy numbers were linked to higher levels of oxidative stress in mammals [70]. The discussed examples for contractions/expansions of *Cyp*, *Gpx*, *Prx*, *cGst*, and *Aqp* in parasites indicate that these gene families are key for the evolution of parasitism as a whole. Therefore, these genes likely play an important role in the adaptive parasite evolution of parasites.

We also provide the first report of *For* orthologs in flatworms, genes linked to behavioural traits in arthropods, nematodes, mammals, and amphibians [15]. Although fecundity, infection intensity [8], and drug resistance [71] in flatworms have been associated with the cGMP-dependent protein kinase (PKG) family, to which the *for*-encoded protein belongs, the function of *For* in these organisms remains obscure. Our analyses indicate that one *For* ortholog has sites under positive selection specific to species of *Cichlidogyrus* from Lake Tanganyika. This observation correlates with the rapid expansion of host-parasites interactions in the lake in recent evolutionary history, but further studies are needed to understand the role of *For* in adaptive evolution [72, 73]. The importance of *For* and PKGs, in general, for other organisms, its potential role in driving infection intensities in parasitic flatworms, and its adaptive evolution in East African species warrant further studies into this gene family to better understand monogenean behavioural genetics.

Lake Tanganyika is a well-known biodiversity hotspot for several animal groups, particularly cichlid fishes. Indeed, the multiple explosive speciation events have made these fishes an established model system in evolutionary biology [74]. We provide the first evidence that their parasites belonging to *Cichlidogyrus* also present unique functional genetic adaptations compared to species of *Cichlidogyrus* elsewhere. Positively selected sites in *Hsp60* and a putative *Hsp40* ortholog of the DnaJ heat shock protein family (HSP40) member A1 (DNAJA1) gene suggest adaptations in the folding/assembly of proteins newly imported into the mitochondria (HSP60) and mitochondrial protein import (DNAJA1) [40] based on functions associated with these genes in other organisms (e.g. humans, *D. melanogaster*). Prior studies already suggested that at least some Lake Tanganyika monogenean lineages have evolved under evolutionary radiations [72, 73]. Although further evidence is needed, the present findings may indicate that this diversification might also be linked to functional adaptations. If true, the cichlid-*Cichlidogyrus* system would represent the first example of a specified genetic adaptation related to an adaptive radiation of flatworms (but see morphological evidence in free-living flatworms from the same lake [75]). To test this hypothesis, evolutionary rates and *Hsp* genetic diversity in more species of *Cichlidogyrus* in and outside of Lake Tanganyika should be analysed.

Species of *Cichlidogyrus* infect an ecologically diverse host lineage, whereas species of *Kapentagyrus* infect a host lineage with a conserved ecological niche (the pelagic zones of rivers and lakes). One may speculate that the gene duplication/loss of stress response genes observed in the *Cichlidogyrus-Kapentagyrus* comparison reflects the contrasting host ecology and evolutionary history and, thus, the adaptive potential of the parasites. Specifically, we identified two potential instances of stress response gene duplication/loss: in comparison with species of *Cichlidogyrus*, species of *Kapentagyrus* lack a gene copy of *Hsp70* (Fig. 5a: group 4b) and *Gstm* (Fig. 5b: *Gstm2a*), and all copies of *Gsto*. If the additional genes in *Cichlidogyrus* indeed resulted from duplication, the additional copies may have increased the adaptive potential to stressful conditions, e.g. their ability to adapt to new environments, as has been described for free-living nematodes [76], fungi [14], and invertebrate groups [77]. In metazoan parasites, prior studies detected gene family expansions in tapeworms [10] and aphids [78], but only rarely are these expansions linked to concrete environmental stressors because of unknown gene functions, but see cases among plant-pathogenic moths [79], and nematodes [13]. However, until a more detailed characterisation of the function of the potentially duplicated genes, the assumption of a role in adaptation to stressful conditions of new environments is hypothetical. Similarly, previous interpretations of expansions of *Hsp70* among closely related lineages as adaptations to environmental stressors, e.g. in tapeworms [10], trypanosomatid protozoans [80], and invasive fishes [81], need to be taken with caution as these expansions were hypothesised to be expressed ‘under certain conditions’ [10] or were only loosely associated with geographical or environmental gradients [80].

No doubt, our study also has conceptual and technological limitations. First, previous studies indicate that copy number evolution can occur between closely related animal species [78] and even strains [80]. No such differences were targeted here because the study focused on differences between lineages not species, an approach taken to avoid mistaking intraspecific allelic gene variants in each pooled DNA samples as paralogs (see the ‘Methods’ section). Future studies might use variant calling pipelines (e.g. [82]) or optimise techniques to sequence genomes from individual specimens to address this problem. The latter approach has recently been successful with monogenean mitochondrial genomes [58]. Another challenge lies in potentially highly divergent sequences that the in silico exon bait capture might fail to detect. Beyond gene copy numbers, we also found that the evolutionary relationships of the gene orthologs sometimes deviated from the evolutionary history of the species (Fig. 5), but this variation might be an artefact of inferring evolutionary histories from small datasets (243–2811 bp) in contrast to multi-gene phylogenies (279 and 78 kb). Furthermore, we only covered nine out of 144 described species of *Cichlidogyrus*. Nevertheless, species of *Cichlidogyrus* constitute a unique study system for host-parasite interactions that combines opportunities to investigate host repertoires and host switching [73], biological invasions [58], and speciation rates [72]. Second, gene models reveal little information on expression patterns. Some genes are only expressed under certain conditions (i.e. inducible genes) and may not be represented in reference transcriptomes [51], e.g. as evidenced for human-infecting flukes [83]. This might explain the absence of several *Hsp70* and *Gpx* transcripts in reference transcriptome used here. Furthermore, environmental stress might not necessarily lead to upregulation (see *Hsp* in Antarctic animals [84, 85]). Therefore, future studies should also aim to quantify gene expression under different environmental conditions using experimental approaches.

## Conclusions

Stress responses are key for the survival of organisms, yet their role in adaptive parasite evolution remains poorly understood. The present study addresses this knowledge gap by analysing the stress response gene presence, copy number variation, and adaptive selection in 11 genomes of two genera of parasitic flatworms. We also resolved the phylogenetic relationships between several lineages of *Cichlidogyrus*, which prior studies using nuclear ribosomal and mitochondrial DNA markers struggled to do [18].

We detected several cases of copy number differences and positively selected gene sites, indicating that alterations in stress response pathways may be a relevant aspect of parasite and disease evolution. Specifically, we highlighted the potential role of mitochondrial protein import and behaviour in parasite of a host lineage under adaptive radiation, which has far-reaching consequences for the many parasite lineages that infect such host lineages (see [86]). Additionally, the absence of cytochrome P450 and kappa and sigma-class glutathione S-transferases in monogenean flatworms is reported, highlighting the unique adaptations monogenean flatworms might present, which warrants further studies into their functional evolution. Consequently, we encourage researchers to not only replicate our approach in other species-rich and functionally diverse lineages but also explore other molecular pathways that might determine adaptive potential and, therefore, the evolution of parasitic diseases.

## Methods

### Sample collection and DNA sequencing

To analyse a representative selection of the species diversity of *Cichlidogyrus*, we collected at least one species from eight of the recently reported 11 main lineages [18] (Additional File 6). Fish hosts were collected as part of previous studies [58, 59] with the help of local fisherfolk and the gills were subsequently extracted from the fishes and stored in absolute ethanol. Individual flatworms were collected from the gills using entomological needles and morphologically identified to species level based on Kuchta [87], Pariselle and Euzet [88], and Vanhove et al. [89]. Morphological identification is considered a reliable method to differentiate species of both target taxa as highlighted by previous studies that highlighted consistency with DNA barcoding approaches [90, 91]. Total genomic DNA extraction was applied on species pools and followed a recently published protocol [60]. For whole-genome amplification, we used the Illustra Ready-To-Go Genomiphi V3 DNA amplification kit (Cytiva, United Kingdom), which was applied to two samples (see Additional File 6). Library preparation (Illumina TruSeq Nano, 350 bp target insert size) and short-read sequencing (151 bp, paired end, HiSeq X) were outsourced to Macrogen Korea (Seoul, South Korea) or Macrogen Europe (Amsterdam, The Netherlands) (for estimated coverages of genomic read-pools, see Additional File 6). Furthermore, we accessed whole-genome sequencing read pools of one species of *Cichlidogyrus* and two of *Kapentagyrus* from previous mitogenomic studies [59, 60] (SRA accessions: https://identifiers.org/insdc.sra:SRX11523770, https://identifiers.org/insdc.sra:SRX18894998, https://identifiers.org/insdc.sra:SRX18894989). We also attempted to use previously published genome short reads of different species of *Cichlidogyrus/Scutogyrus* [92, 93]. However, the coverage of these reads proved to be too low for capturing gene sequences targeted here. Raw sequence reads were trimmed through *Trimmomatic* v0.39 [94] using a sliding window approach (settings: *SLIDINGWINDOW:4:28 HEADCROP:5 MINLEN:100 ILLUMINACLIP:TruSeq3-PE.fa:2:30:10:2:True*). The quality of filtered reads was checked in *FastQC* v0.11.8 [95]. Raw Illumina reads generated as part of this study were submitted to the NCBI Sequencing Read Archive (SRA) (accession numbers: https://identifiers.org/insdc.sra:SRR31400484–https://identifiers.org/insdc.sra:SRR31400491) under BioProject accession https://identifiers.org/ncbi/bioproject:PRJNA1186934.

### Gene selection for species tree estimation and stress response genes

We used single copy ortholog genes to infer the phylogenetic backbone (species tree) of the parasite species. To date, nuclear ribosomal genes (28S and 18S rDNA and the internal transcribed spacers) and mitochondrial genes have been used as phylogenetic markers across most animal taxa as their multi-copy nature increases the likelihood of successful amplification of the target loci [96]. However, both rDNA and mitochondrial DNA have high substitution rates in flatworms [97] that may cause sequence alignment errors, which in return create noise in phylogenetic analyses (e.g. long-branch attraction of rapidly evolving lineages). Genome data provide a large number of alternative markers for phylogenetic inference, e.g. large datasets composed of single-copy orthologous genes can be used to resolve phylogenetic relationships. Recently, this single-copy ortholog approach has been adopted for a neodermatan phylogeny [20] including several monogenean species—one of them belonging to *Scutogyrus* Pariselle & Euzet, 1995, a nested lineage within *Cichlidogyrus*. The resulting phylogeny was based on 137 and 479 orthologous groups of proteins inferred from the *BUSCO* v4 [29] and *OMA* v2.6.0 [98] pipelines, respectively. As *S. longicornis* is a member of one of the target lineages of this study, we used the previously assembled single copy protein sequences [20] as bait sequences for the downstream putative protein sequence assembly (Fig. 2a, single-copy orthologs). The assembly of the *BUSCO* and *OMA* genes through the pipeline *HybPiper* v2.0 [99] may also include genes other than single-copy genes in genomes of species of *Cichlidogyrus*, but this was considered unlikely because these conserved genes have previously been demonstrated to have only a single copy in *S. longicornis* and other flatworm lineages [20].

For the stress genes, we focused on 12 gene families from three different functional groups: antioxidant enzymes (*Cyp*, *Gpx*, *Mgst*, *cGst*, *Gstk*, *Prx*, *Sod*, and *Tgr*), heat shock proteins (*Hsp10*, *Hsp40*, *Hsp60*, *Hsp70*, and *Hsp90*), and *foraging* orthologs (see Fig. 2 and Additional File 3 for abbreviations). For *cGst*, we targeted all known gene families and classes [100]. For the other antioxidant enzymes, we included the main groups previously reported from parasitic flatworms [55]. For *Hsp*, we included the gene families investigated extensively in flatworms in a recent study [9]. An illustration of the ortholog selection process described below can be found in Fig. 6. As performance of exon bait capture (see section below: *DNA sequence assembly*) decreases with phylogenetic distance, we aimed to use bait sequences from species that are as closely related as possible to the target taxa. However, nucleotide and amino acid sequences of the targeted genes have rarely been explicitly targeted in genome assemblies of monogenean flatworms (except for the *Hsp70* subfamily in *Gyrodactylus salaris* Malmberg, 1957 [11]). Therefore, we compiled a set of previously published protein sequences of other flatworm groups (Additional File 3). As no *for* orthologs have been reported in flatworms in previous studies, we included protein sequences of *for* isoforms of *D. melanogaster*. All bait sequences were used to detect putative protein orthologs in a draft annotation of a genome assembly of *Cichlidogyrus casuarinus* (Additional File 3; Fig. 2a, stress genes) (WGS accession JBJKFK000000000) using *BLAST* + v2.13.0 [101] (Fig. 1b). These data were selected for being the only available genome annotation of a species of *Cichlidogyrus*, which is currently being optimised as part of a separate study. For gene families, for which we did not detect orthologs in *C. casuarinus*, we used the initial non-monogenean search sequences as baits (Additional File 3) and also verified the potential absence of these genes through a *BLAST* search of published monogenean genomes in NCBI GenBank [11, 32, 56, 57]. The assembly and annotation process of the draft genome of *C. casuarinus* is detailed in Additional File 2. For a list of accession numbers and the respective protein IDs in *C. casuarinus*, see Additional File 3. A total of 48 putative protein sequences of *C. casuarinus* with query coverages above 90% were considered highly likely to represent genuine orthologs and included as bait in downstream analyses. In case of multiple hits, all sequences were included as baits as they might present potential duplications. Following the selection procedure for the and stress genes, we used 479 (OMA) and 137 (BUSCO) bait sequences for single-copy orthologs and 48 for the stress genes.

### DNA sequence assembly and paralog filtering

Target genes in individual samples were identified through an in-situ exon bait capture approach as implemented in the pipeline *HybPiper* v2.0 [99]. *HybPiper* uses a bait file to map the trimmed paired-end and unpaired reads of all analysed species against the bait sequences (Fig. 2c). The bait file for the single-copy orthologs were compiled as detailed above. The bait file for the stress genes was compiled through the sequences of *C. casuarinus*. For the target files of both the stress genes and the single-copy orthologs, we used the protein sequences rather than the nucleotides sequences as gene assemblies reportedly improve when using the former [99]. In *HybPiper*, we used default parameters for the assembly, contig alignment and stitching process, and flagging of potential paralogs (the contig with highest read depth is selected as ‘main hit’) and chimeric sequences, but we chose *DIAMOND* v2.0.15 for the rapid alignments of sequencing reads [48].

The pooling approach during the sample acquisition means that paralogs flagged by *HybPiper* may be both orthologs in the sampled population or ‘real’ paralogs. To exclude the former and to remove contaminant sequences (e.g. host DNA, microorganisms associated with host gills or flatworm parasites), we manually curated sequences by performing five filtering steps (Fig. 2d), one step for the single-copy orthologs (i) and four steps for the stress genes (ii–iv):

(i) We excluded any single-copy ortholog alignments for which paralogs were flagged in *HybPiper* to minimise the risks of accidentally including any contaminant sequences. We also excluded single-copy ortholog alignments for which sequences were not recovered from all 11 target species to minimise the impact of missing data on the species tree.
(ii) We applied a *BLAST* + search to all assembled protein sequences of stress genes against the NCBI protein database to exclude contaminants. Best-hit sequences with > 90% identity with non-flatworm sequences were excluded.
(iii) We performed phylogenetic analyses with all potential paralogous sequences of the stress genes in the *HybPiper* output (see details below) and identified groups of sequences with a lowest common ancestor (LCA) (i.e. the common ancestor furthest away from the root) [102] as orthologous groups. If these groups of sequences (i.e. potential orthologous groups) included sequences from all target species, they were immediately assembled into gene alignments for downstream analyses, using the main hits assembled by *HybPiper*. Groups for which genes were only recovered for some target species were subjected to a second run in *HybPiper* to detect gene orthologs. A second phylogenetic analysis was applied to the resulting protein alignments combined with the sequences assembled in the first *HybPiper* run from the same gene family. The sequences from the second run were again filtered through the LCA approach. In cases for which *HybPiper* did not provide a main hit, the paralog sequence with the maximum read depth was retained in each orthologous group, supplanting the missing hit. This selection by read depth might create a bias but is done to minimise the effects of sequencing errors at lower read depths.
(iv) We excluded orthologous groups of stress genes detected in less than three target species to further minimise the effects of variation in the sampled populations of each DNA read pool.
(v) We checked whether alignments of stress gene models not targeted with the bait sequences (paralogs suggested by *HybPiper*) represented fragments of other assembled gene models using *BLAST* + and excluded such truncated sequences.

Following the filtering steps, we inferred functional descriptions and gene ontology (GO) classes for each orthologous group using *PANNZER2* [103] (Fig. 2e). These GO terms were only considered reliable if the annotations were assigned to orthologs of three or more species. We also verified the presence of the gene sequences through a *BLAST* + search (*tblastn*) against a recently published transcriptome annotation of *S. longicornis* [51], interpreting sequence identities and query coverage > 95% as confirmatory of transcription (Fig. 2e). Alignments of exon sequences can be accessed at Zenodo ( 10.5281/zenodo.14236484).

### Phylogenetic analyses

We performed phylogenetic analyses for three different sequence datasets: species trees (based on single-copy orthologs, 277 BUSCO and 86 OMA loci, respectively), gene family trees for sequence filtering and paralog identification (e.g. *Gst* and *Hsp70*), and gene trees (for each of the 48 groups of orthologs for the targeted gene families). Phylogenetic analyses of the nucleotide sequences were performed under the maximum likelihood (ML) criterion. Sequences of all genes were aligned and trimmed with codon awareness through *MACSE* v2.06 using the options *trimNonHomologousFragments*, *alignSequences*, and *trimAlignment* [104, 105]. For the gene family trees, we did not trim the alignments as many informative sites would be removed due to high divergence between genes of the same gene family. Codon substitution models were selected by gene through *ModelFinder* in *IQ-Tree* [106]. We estimated tree topologies through *IQ-Tree* v2.2.0 [107, 108], estimating branch support through ultrafast bootstraps [109] and Shimodaira-Hasegawa–like approximate likelihood ratio tests (SH-aLRT) [110] with 10,000 replicates. We considered nodes with an ultrafast bootstrap value (UF-Boot) ≥ 95 and an SH-aLRT statistic ≥ 80 as well-supported. Phylogenetic trees were visualised through *ggtree* v3.6.2 [111, 112] in *R* v4.3.2 [113].

### Comparison of gene vs. species tree topologies

We employed two approaches to assess topological differences of the species tree, the gene family trees (oxidative stress, heat shock, aquaporin, and *foraging* genes), and the single gene trees: visual inspection and multidimensional scaling. First, we assessed the phylogenies of the gene families qualitatively through visual inspection to detect potential deletions and/or duplications of genes among the parasite species investigated here. We followed an approach based on the LCA (see above), where nodes are either considered speciation or duplication nodes [102] according to parsimony criteria (reconciliation). Groups of single sequences from different species that formed monophyletic clades were considered orthologous.

In the second step, we tested whether the tree topologies of each orthologous group of stress response genes deviated from the species trees of *Cichlidogyrus* using multi-dimensional scaling (MDS) based on Kendall-Colijn distances of the trees [114]*.* This analysis was performed to infer whether the evolution of the target genes showed concordance with the evolution of the lineage (species tree). To detect topological differences of gene trees regarding *Cichlidogyrus*, sequences of *Kapentagyrus* were dropped from these trees using the function *drop.tip* in the *R* package *ape* v5.7–1 [115]. All gene trees with missing taxa (less than nine species of *Cichlidogyrus*) were also excluded as MDS requires complete datasets. Finally, we performed the MDS analysis through the package *treespace* v1.1.4.2 [116] on all 43 remaining gene trees.

### Positive selection of gene sites

To detect signals of adaptive evolution in the stress response genes, we analysed patterns of synonymous and non-synonymous changes (d_N_/d_S_) in each of the 48 sequence alignments. We tested (I) whether stress genes of *Cichlidogyrus* and *Kapentagyrus* present gene sites that show patterns of positive selection (d_N_/d_S_ > 1) and (II) if positively selected sites were more prevalent in certain clades/species (branch-site tests). Specifically, we tested if stress genes of species of *Cichlidogyrus* outside of Lake Tanganyika have undergone positive selection (IIa) and if stress genes of East African species of *Cichlidogyrus* infecting hosts that have undergone adaptive radiation (Lake Tanganyika clade, Fig. 3) have done so (IIb). These codon analyses were performed in *CODEML* in *PAML* v4.10 [53] using the OMA-based species tree (for its higher node support, see Fig. 3 and Additional File 1) and the average nucleotide frequencies at the three codon positions (*CodonFreq* = *2*).

For (I), we performed pairwise likelihood ratio tests (LRTs) between models (M) with heterogeneous d_N_/d_S_ across sites: M1a vs. M0 (rate heterogeneity), M2a vs. M1a (positive selection, test 1), and M8 vs. M7 (positive selection, test 2). The rate heterogeneity test serves to test variability in selective pressure across sites. The other two tests serve to detect positive selection. If both tests were positive, we considered this confirmation of strongly positively selected sites. If only M8 vs. M7 turned out positive, we interpreted this result as a sign of the presence of weakly (yet significantly) positively selected sites as the second test is less stringent [117].

For (II), we performed pairwise LRTs between models with heterogeneous d_N_/d_S_ across sites and clades. In accordance with *PAML* guidelines [117], the clades with hypothesised positively selected sites were defined as foreground branches, and two models were applied to each case: M1 (site model M2a, see above) and M0 (site model M2a, but with d_N_/d_S_ fixed to 0). We tested two selected clades based on our hypotheses: *Cichlidogyrus* without Lake Tanganyika (IIa) and *Cichlidogyrus*–Lake Tanganyika only (IIb). If LRTs were positive, we considered positively selected sites to be present in the tested clades. For all tests (I, IIa, IIb), we also performed a robustness analysis by varying the *CodonFreq* parameter (0, 1, 2, 3) and assessing differences in the outcome [53].

## Supplementary Information

Additional file 1. Species tree of *Cichlidogyrus* and *Kapentagyrus* inferred from 68 single-copy orthologs based on a subset of genes selected by Caña-Bozada et al. (2023), who used the BUSCO pipeline. Support values: ultrafast bootstraps (UF-Boot)/Shimodaira-Hasegawa-like approximate likelihood ratio tests (SH-aLRT) (see the ‘ Methods ’ section), asterisks (*) indicate support below threshold (UF-Boot ≤ 95, SH-aLRT ≤ 80). Abbreviations: Ccas– *Cichlidogyrus casuarinus* , Ccir– *C. cirratus*, Chal– *C. halli*, Ckap– C. sp. ‘kapembwa’, Cscl– *C. sclerosus*, Cthu– *C. thurstonae* , Ctil– *C. tilapiae*, Czam– *C. zambezensis*, Slon– *Scutogyrus longicornis*, Klim– *Kapentagyrus limnotrissae*, Ktan– *K. tanganicanus*. Scale bar: estimated number of substitutions per site.

Additional file 2. Draft assembly and annotation of genome of *Cichlidogyrus casuarinus*.

Additional file 3. Overview of sequences used for bait capture of target gene groups and baited sequences (hits) with annotations. Heat shock protein sequences can be accessed using the protein IDs [9] at *UniProt* [43]. Annotations were inferred from PANNZER2 [77] (see Fig. 2 ).

Additional file 4. Number of paralogs by parasite species flagged for each of the protein bait sequences of *Cichlidogyrus casuarinus* (see Supplementary File S2) for stress gene assembly. Gene names reflect bait sequences from draft annotation ( *C. casuarinus* ) and not the final assembled genes. Abbreviations: Ccas– *Cichlidogyrus casuarinus*, Ccir– *C. cirratus*, Chal– *C. halli*, Ckap– C. sp. ‘kapembwa’, Cscl– *C. sclerosus*, Cthu– *C. thurstonae*, Ctil– *C. tilapiae*, Czam– *C. zambezensis*, Slon– *Scutogyrus longicornis*, Klim– *Kapentagyrus limnotrissae*, Ktan– *K. tanganicanus*.

Additional file 5. Stress gene tree topologies produced under the maximum likelihood criterion. Abbreviations: Ccas–*Cichlidogyrus casuarinus*, Ccir–*C. cirratus*, Chal–*C. halli*, Ckap–C. sp. ‘kapembwa’, Cscl–*C. sclerosus*, Cthu–*C. thurstonae*, Ctil–*C. tilapiae*, Czam–*C. zambezensis*, Slon–*Scutogyrus longicornis*, Klim–*Kapentagyrus limnotrissae*, Ktan–*K. tanganicanus*.

Additional file 6. Sampling data of collected specimens including reference for sampling campaigns and published whole-genome sequencing data.

Additional file 7. Extended version of Fig. 4. Detected putative stress gene orthologs (protein sequences) including species coverage (cyan = *Cichlidogyrus*, red = *Kapentagyrus*) (a), gene ontology (GO) terms (black = term applies) (b), presence in transcriptome annotation (blue = present) (c), and hypothesis testing of different models for detecting positively selected gene sites (I, IIa, IIb) (d) with * indicating *P* < 0.05 for test results and the colour scale indicating the likelihood ratio test statistics (LRT) (see the ‘Methods’ section). Rows and columns of the GO heatmap are automatically sorted through Euclidean distances as implemented in ComplexHeatmap.

## Data Availability

Raw Illumina reads were submitted to the NCBI Sequencing Read Archive (SRA) (accession numbers: https://identifiers.org/insdc.sra:SRR31400484–https://identifiers.org/insdc.sra:SRR31400491) under BioProject accession https://identifiers.org/ncbi/bioproject:PRJNA1186934. This Whole Genome Shotgun project has been deposited at DDBJ/ENA/GenBank under the accession https://identifiers.org/ncbigene:JBJKFK000000000. The version described in this paper is version https://identifiers.org/ncbigene:JBJKFK000000000. Phylogenetic trees and alignments were deposited in Zenodo (https://www.doi.org/10.5281/zenodo.14236484).

## Abbreviations

*Aqp*: Aquaporin
*BiP*: Binding protein
BUSCO: Benchmarking Universal Single-Copy Orthologs
cGMP: Cyclic guanosine monophosphate
*cGst*: Cytosolic glutathione S-transferase
*Cyp*: Cytochrome P450
d_N_/d_S_: Ratio of non-synonymous to synonymous substitutions
DNAJA1: DnaJ heat shock protein family (Hsp40) member A1
*For*: Foraging
GO term: Gene ontology term
*Gpx*: Glutathione peroxidase
*Gst*: Glutathione S-transferase
*Gsta*: Glutathione S-transferase alpha-class
*Gstk*: Glutathione S-transferase kappa-class
*Gstm*: Glutathione S-transferase mu-class
*Gsto*: Glutathione S-transferase omega-class
*Gstp*: Glutathione S-transferase pi-class
*Gsts*: Glutathione S-transferase sigma-class
*Gstz*: Glutathione S-transferase zeta-class
*Hsp10*: Heat shock 10 kDa protein
*Hsp40*: Heat shock 40 kDa protein
*Hsp60*: Heat shock 60 kDa protein
*Hsp70*: Heat shock 70 kDa protein
*Hsp90*: Heat shock 90 kDa protein
*Hyou1*: Hypoxia upregulated protein 1
LCA: Lowest common ancestor
LRT: Likelihood ratio test
MDS: Multidimensional scaling
*Mgst*: Microsomal glutathione S-transferase
OMA: Orthologous MAtrix
PKG: CGMP-dependent protein kinase or protein kinase G
*Prx*: Peroxiredoxin
SH-aLRT: Shimodaira-Hasegawa approximate likelihood ratio test
*Sod*: Superoxide dismutase
Tgr: Thioredoxin glutathione reductase
UF-Boot: Ultrafast bootstrap
