# Supplementary figures and images for: Adaptive evolution of stress response genes in parasites aligns with host niche diversity
Source: BMC Biol. 2025 Jan 13;23:10. doi: 10.1186/s12915-024-02091-w (PMC11727194; doi:10.1186/s12915-024-02091-w)

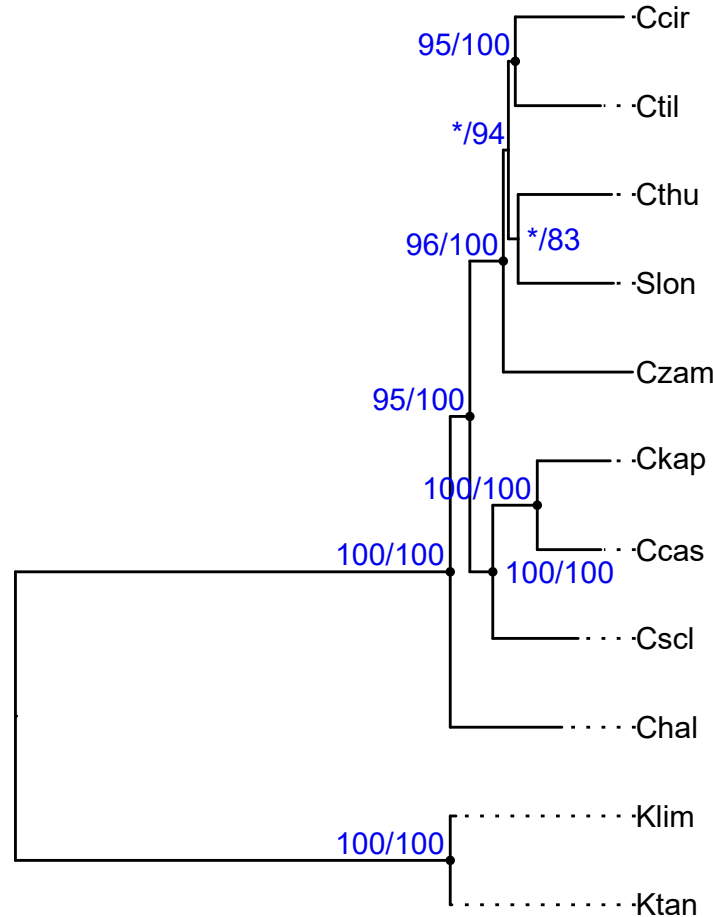

0.4

Supplement: Supplementary file 1 — Additional file 1. Species tree of Cichlidogyrus and Kapentagyrus inferred from 68 single-copy orthologs based on a subset of genes selected by Caña-Bozada et al. (2023), who used the BUSCO pipeline. Support values: ultrafast bootstraps (UF-Boot)/Shimodaira-Hasegawa-like approximate likelihood ratio tests (SH-aLRT) (see the ‘ Methods ’ section), asterisks (*) indicate support below threshold (UF-Boot ≤ 95, SH-aLRT ≤ 80). Abbreviations: Ccas– Cichlidogyrus casuarinus , Ccir– C. cirratus, Chal– C. halli, Ckap– C. sp. ‘kapembwa’, Cscl– C. sclerosus, Cthu– C. thurstonae , Ctil– C. tilapiae, Czam– C. zambezensis, Slon– Scutogyrus longicornis, Klim– Kapentagyrus limnotrissae, Ktan– K. tanganicanus. Scale bar: estimated number of substitutions per site. [file 12915_2024_2091_MOESM1_ESM.pdf]

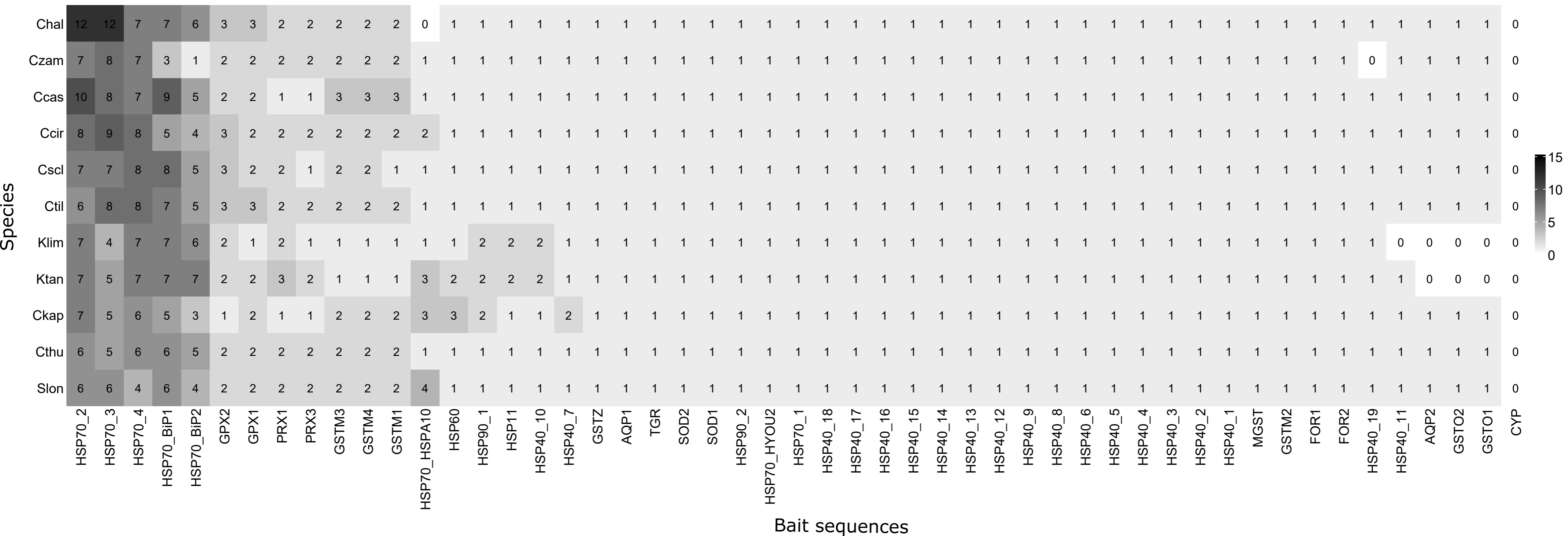

Supplement: Supplementary file 4 — Additional file 4. Number of paralogs by parasite species flagged for each of the protein bait sequences of Cichlidogyrus casuarinus (see Supplementary File S2) for stress gene assembly. Gene names reflect bait sequences from draft annotation ( C. casuarinus ) and not the final assembled genes. Abbreviations: Ccas– Cichlidogyrus casuarinus, Ccir– C. cirratus, Chal– C. halli, Ckap– C. sp. ‘kapembwa’, Cscl– C. sclerosus, Cthu– C. thurstonae, Ctil– C. tilapiae, Czam– C. zambezensis, Slon– Scutogyrus longicornis, Klim– Kapentagyrus limnotrissae, Ktan– K. tanganicanus. [file 12915_2024_2091_MOESM4_ESM.pdf]

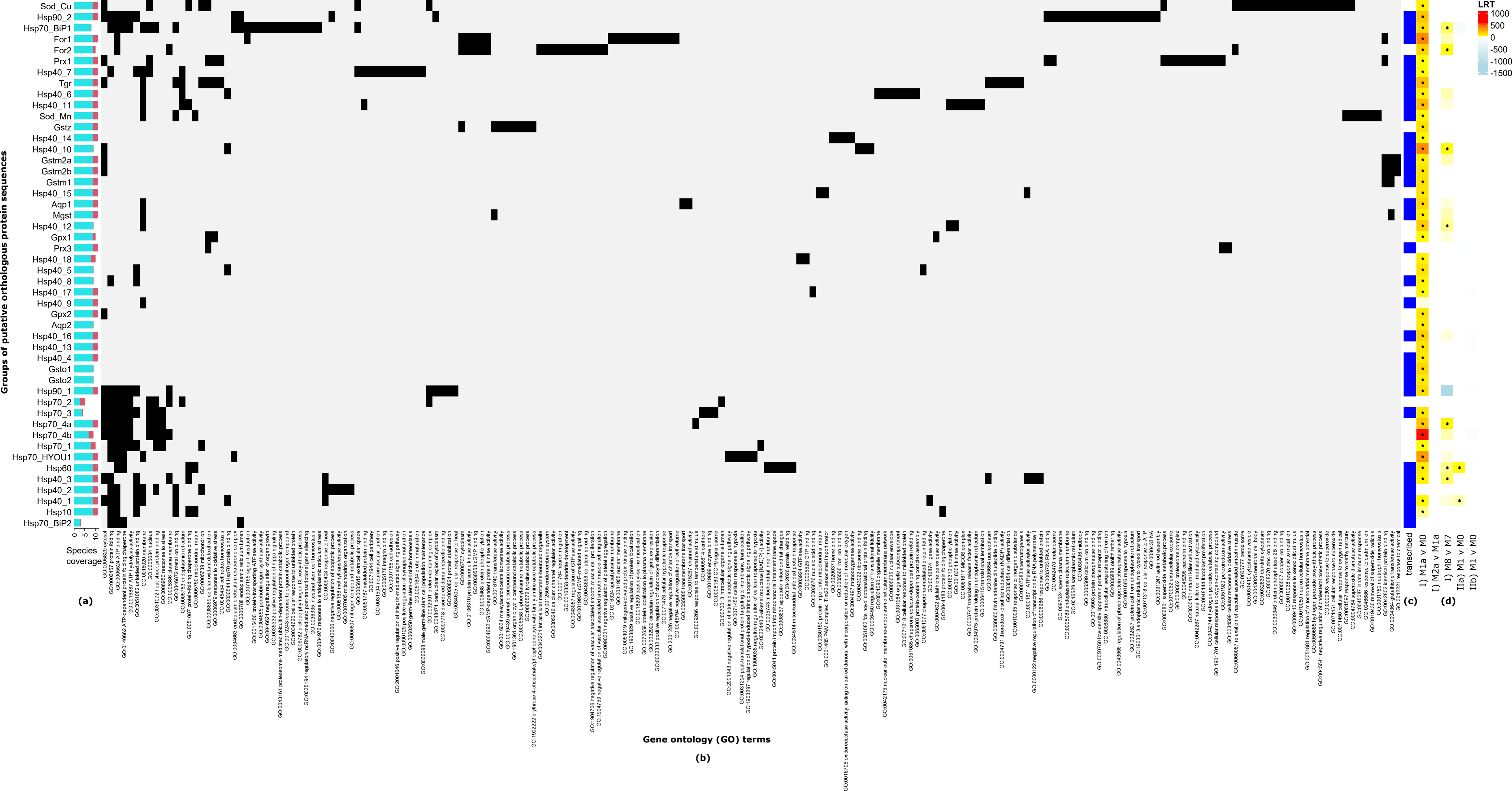

Supplement: Supplementary file 7 — Additional file 7. Extended version of Fig. 4. Detected putative stress gene orthologs (protein sequences) including species coverage (cyan = Cichlidogyrus, red = Kapentagyrus) (a), gene ontology (GO) terms (black = term applies) (b), presence in transcriptome annotation (blue = present) (c), and hypothesis testing of different models for detecting positively selected gene sites (I, IIa, IIb) (d) with * indicating P < 0.05 for test results and the colour scale indicating the likelihood ratio test statistics (LRT) (see the ‘Methods’ section). Rows and columns of the GO heatmap are automatically sorted through Euclidean distances as implemented in ComplexHeatmap. [file 12915_2024_2091_MOESM7_ESM.pdf]
