# Supplementary material for: Adaptive evolution of stress response genes in parasites aligns with host niche diversity
Source: BMC Biol. 2025 Jan 13;23:10. doi: 10.1186/s12915-024-02091-w (PMC11727194; doi:10.1186/s12915-024-02091-w)
Supplement: Supplementary file 2 — Additional file 2. Draft assembly and annotation of genome of Cichlidogyrus casuarinus. [file 12915_2024_2091_MOESM2_ESM.pdf]

Supplementary File S2. Draft assembly and annotation of genome of *Cichlidogyrus casuarinus*

DNA extraction and library preparation followed procedures previoulsy described [21]. In brief, genomic DNA was extracted using the Quick-DNA™ Miniprep Plus Kit (Zymo Research, Irvine, CA, USA) following the manufacturer's instructions with minor modifications, specifically, initial incubation overnight, and elution in 2 × 50 µL after 10 min incubation at room temperature each. DNA quantity was assessed using a Qubit 4.0 fluorometer and the Qubit dsDNA BR Assay. DNA integrity was assessed on an Agilent TapeStation system. Library preparation (Illumina Nextera XT, 550 bp insert size) and sequencing on the NovaSeq6000 (2× 150 bp) platform were outsourced (Macrogen Europe, The Netherlands).

Illumina reads were quality checked using FastQC and adapter and quality trimmed using Trimgalore v. 0.6.0 (https://github.com/FelixKrueger/TrimGalore; accessed 11 Apr 2024), which employs Cutadapt [22] for adapter trimming. Subsequently, error correction was performed using correction module of SPAdes v3.14.0 [23]. Read pairs were merged using USEARCH v11.0.667_i86linux32 [24]. Genome assemblies were performed using different sets of reads, specifically, trimmed, corrected, and merged (with trimmed and corrected reads), using SPAdes, AbySS v2.2.5 [25], and Platanus v1.2.4 [26]. The kmer lengths used for ABySS were selected using KmerGenie v1.7.051 [27]. The contiguity of all assemblies was assessed using Quast-LG v5.0.2 [28]. Assembly completeness was evaluated using BUSCO v. 5.2.1_cv1 [29] (dataset eukaryota_odb10) and potential host contamination was assessed using BlobTools v1.1.1 [30]. All assemblies were then compared with respect to contiguity (N50), BUSCO completeness and contamination. The assembly result obtained with Platanus using corrected reads merged with usearch was selected as the best for subsequent analyses based on these three criteria. The entire process (data trimming, correction, merging, assembly, assembly evaluation) was run through the workflow demogenas (https://github.com/chrishah/demogenas; accessed 11 Apr 2024) implemented with Snakemake [31].

The draft genome of *Cichlidogyrus casuarinus* was annotated following a strategy previoulsy described (see Vorel et al. (2023)), with some modifications. In brief, core eukaryotic genes were identified in the final assembly using CEGMA v2.5 [33] and BUSCO v3.0.2 [34] (Metazoa dataset, odb9, 978 searched groups). The latter was run with the *optimize_augustus* option to train the AUGUSTUS v3.3.3 *ab initio* gene predictor [35] in the process. Genes identified by CEGMA were used to train the SNAP v2006–07-28 [36] *ab initio* gene predictor. Species-specific repeats were identified

using RepeatModeler v1.0.10 [37]. RepeatMasker v4.0.7 [38] was then run to mask repetitive regions, using 1) the de novo library identified in the previous step, and 2) using a prebuilt repeat library (RepBaseRepeatMaskerEdition-20181026) with species set to *eukaryota*. *Ab-initio* gene predictor Genemark-ES [39] (*gmes_petap.pl*) v4.69_lic was run on the repeat soft-masked genome. As protein evidence that would further inform downstream gene prediction, we concatenated the complete UniProt/Swiss-Prot protein database [40] (release 2022_01) and 33 available protein complements of parasitic flatworms downloaded from the NCBI GenBank [41] and WormBase ParaSite databases [42] (accessed 2 Feb 2022). To remove redundancy in the reference protein set, it was clustered at 98% similarity using CD-HIT [43] v4.8.1. Further, gene prediction was performed in two passes: First, using MAKER2 [44] v2.31.10 on the repeat masked genome, based on the physical protein (see above), and using the gene models obtained with SNAP (see above). Gene models of the first MAKER pass (only genes with evidence score < 0.1) were used to retrain the AUGUSTUS and SNAP ab-initio predictors. In a second pass, MAKER2 was rerun combining all evidence and using AUGUSTUS, Genemark, and SNAP and their pre-trained models. Subsequently we ran the *predict* Funannotate v1.8.7 (https://github.com/nextgenusfs/funannotate, accessed 31 Jan 2023) with AUGUSTUS, SNAP, and GlimmerHMM [43], incorporating the gene models initially predicted with Genemark and predictions obtained via the two passes of MAKER (weight 2). The resulting set of gene predictions was functionally annotated using the annotation module of Funannotate, combining the results from InterProScan [45] v5.48–83.0 with a similarity search against databases UniProt/Swiss-Prot (release 2022_01), MEROPS[46] (database of proteolytic enzymes and inhibitors, release 12.0), and Phobius [47] using search tool DIAMOND [48] v2.0.7 (BLASTp algorithm) and with a search against the complete eggNOG 5.0 database [49] conducted with the eggNOG-mapper [50] (emapper.py) v. 1.0.3. The entire prediction and annotation process as described above was run through Annocomba (https://github.com/reslp/annocomba, accessed 31 Jan 2023), which uses the Snakemake workflow management system [31].
