# Supplementary material for: Adaptive evolution of stress response genes in parasites aligns with host niche diversity
Source: BMC Biol. 2025 Jan 13;23:10. doi: 10.1186/s12915-024-02091-w (PMC11727194; doi:10.1186/s12915-024-02091-w)
Supplement: Supplementary file 3 — Additional file 3. Overview of sequences used for bait capture of target gene groups and baited sequences (hits) with annotations. Heat shock protein sequences can be accessed using the protein IDs [9] at UniProt [43]. Annotations were inferred from PANNZER2 [77] (see Fig. 2 ). [file 12915_2024_2091_MOESM3_ESM.pdf]

Additional File 3. Overview of sequences used for bait capture of targeted stress gene groups and baited sequences (hits) with annotations. Heat shock protein sequences can be accessed using the protein IDs [9] at *UniProt* [40]. Annotations were inferred from *PANNZER2* [103] (see Fig. 2).

**Bait sequence**				**Hits**		**Annotations**
Name	Family	Species	*GenBank* Accession	Protein ID (*C. casuarinus*)	*HybPiper*	Description
Cytochrome P450 (CYP)		*Schistosoma mansoni*<br>*Opisthorchis felineus*<br>*Echinococcous multilocularis*		—	—	—
Glutathione peroxidase (GPX)		*S. mansoni*	Q00277.2	Ciccas_004298-T1	Group 1: all but *K. limnotrissae*	Glutathione peroxidase
				Ciccas_013506-T1	Group 2: all	Glutathione peroxidase
Glutathione *S*-transferase (GST)	Microsomal (MGST)	*E. multilocularis*	CDS61239	Ciccas_005779-T1	All	Microsomal glutathione S-transferase 3
	Alpha-class (GSTA)	*Ciona intestinalis*	XP_002119784.1<br>XP_002120526.1<br>XP_009861494.1	—	—	—
	Zeta-class (GSTZ)	*Clonorchis sinensis*	GAA48819.1	Ciccas_005965-T1	All but *K. limnotrissae*	Maleylacetoacetate isomerase
	Mu-class (GSTM)	*E. multilocularis*	CDS58059.1<br>CDS58060.1<br>CDS58083.1<br>CAA59739.1	Ciccas_002592-T1	Group 1: all	Glutathione S-transferase class-mu 26 kDa isozyme
			CDS58090.2<br>CDS58091.1<br>CDS58092.1<br>CDS58086.2<br>CDS58094.2<br>CDI96481.2	Ciccas_001521-T1<br>Ciccas_010945-T1<br>Ciccas_008177-T1	Group 2a: *Cichlidogyrus* spp. without *C. sclerosus*	Glutathione transferase
					Group 2b: all	Glutathione transferase
	Pi-class (GSTP)	*Homo sapiens*<br>*Danio rerio*	NP_000843.1<br>NP_001156323.1	—	—	—
	Sigma-class (GSTS)	*E. multilocularis*	CDS59356.1<br>CDS57347.1	—	—	—
		C. sinensis	GAA52095.1<br>GAA33791.2<br>AAD17488.1<br>GAA54850.1<br>GAA54851.1	—	—	—
	Omega-class (GSTO)	*C. sinensis*	GAA51230.1<br>GAA51230.1	Ciccas_008546-T1	*Cichlidogyrus* spp.	unknown
				Ciccas_005584-T1	*Cichlidogyrus* spp.	unknown
	Mitochondrial kappa-class (GSTK)	*C. sinensis*		—	—	—
Peroxiredoxin (PRX)	1	*S. mansoni*	AAD17299.1	Ciccas_000490-T1		Peroxiredoxin

	2	*S. mansoni*	XP_018645129.1	—	—	—
	3	*S. mansoni*	AAG15506.1	Ciccas_012151-T1		Thioredoxin-dependent peroxide reductase, mitochondrial
Superoxide dismutase (SOD)	Cu-Zn	*S. mansoni*	Q01137.1	Ciccas_003175-T1		Superoxide dismutase [Cu-Zn]
	Mn	*Schistosoma japonicum*	AAW26480.1	Ciccas_004492-T1		Superoxide dismutase
Thioredoxin glutathione reductase (TGR)		*S. mansoni*	XP_018649018.1	Ciccas_000084-T1		thioredoxin-disulfide reductase
Heat shock protein 10 kDa		*S. mansoni*	XP_018653498.1	Ciccas_004103-T1		10 kDa heat shock protein, mitochondrial
Heat shock protein 40 kDa	31 members	*S. mansoni*	see [9]	Ciccas_008900-T1	Group 1	DnaJ homolog subfamily A member 1
				Ciccas_002633-T1	Group 2	Tumorous imaginal discs, mitochondrial
				Ciccas_001763-T1	Group 3	DnaJ heat shock protein family (Hsp40) member B4
				Ciccas_007936-T1	Group 4	J domain-containing protein
				Ciccas_006109-T1	Group 5	DnaJ-like protein subfamily B member 8
				Ciccas_001787-T1	Group 6	DnaJ heat shock protein family (Hsp40) member B14
				Ciccas_006479-T1	Group 7	DnaJ homolog shv/DnaJ homolog subfamily B member 11
				Ciccas_001842-T1	Group 8	unknown
				Ciccas_000913-T1	Group 9	DnaJ homolog subfamily C member 1
				Ciccas_002375-T1	Group 10	DnaJ homolog subfamily C member 2
				Ciccas_004035-T1	Group 11	DnaJ homolog subfamily C member 3/putative dsrna-activated protein kinase inhibitor p58
				Ciccas_001896-T1	Group 12	DnaJ heat shock protein family (Hsp40) member C7
				Ciccas_009179-T1	Group 13	DnaJ homolog subfamily C member 8
				Ciccas_004467-T1	Group 14	DnaJ homolog subfamily C member 9
				Ciccas_012246-T1	Group 15	Mitochondrial import inner membrane translocase subunit TIM14
				Ciccas_000761-T1	Group 16	unknown
				Ciccas_009006-T1	Group 17	DnaJ subfamily C member 17
				Ciccas_009734-T1	Group 18	DnaJ sub C member 27, variant 2
Heat shock protein 60 kDa	HSP60	*S. mansoni*	XP_018645622.1	Ciccas_000395-T1		Heat shock protein 60
Heat shock protein 70 kDa	HSPA9 protein-like	*S. mansoni*	see [9]	Ciccas_010128-T1	Group 1: All except *C. halli* and *C.* sp. 'kapembwa'	Heat shock protein cognate 5
					Group 1bis:	Heat shock protein 70

					All except *C. halli* and *C.* sp. 'kapembwa'	
	Hypoxia up-regulated protein 1 precursor-like	*S. mansoni*	see [9]	Ciccas_003925-T1	Group HYOU1:<br>All	Molecular chaperone grp170/sil1 *Hsp*70 superfamily protein + Hypoxia up-regulated protein 1
	Endoplasmic reticulum chaperone BiP precursor-like	*S. mansoni*	XP_018649109.1	Ciccas_002227-T1	Group BiP1:<br>*Cichlidogyrus* spp. except *C. halli*	Heat shock 70 kDa protein cognate 3
				Ciccas_002546-T1	Group BiP2:<br>*C. casuarinus*<br>*C. halli*<br>*S. longicornis*	Heat shock 70 kDa protein cognate 3
	Others (4 members)	*S. mansoni*	see [9]	Ciccas_008712-T1<br>Ciccas_006072-T1<br>Ciccas_007222-T1<br>Ciccas_012130-T1	Group 2:<br>*C. casuarinus*<br>*C. halli*<br>*S. longicornis*<br>*Kapentagyrus* spp.	Heat shock protein 70
					Group 3:<br>*C. casuarinus*<br>*C. cirratus*<br>*C. sclerosus*<br>*C. thurstonae*<br>*C. tilapiae*<br>*C. zambezensis*<br>*S. longicornis*	Heat shock protein 70
					Group 4a:<br>All	Heat shock protein 70
					Group 4b:<br>*Cichlidogyrus* spp.	Heat shock protein 70
Heat shock protein 90 kDa	HSP 90-alpha isoform 2-like	*S. mansoni*	see [9]	Ciccas_003030-T1	Group 1: all	Heat shock protein 83
				Ciccas_005168-T1	Group 2: all	Endoplasmin
	TRAP1-like	*S. mansoni*	XP_018652104.1	—	—	—
	Endoplasmin precursor-like	*S. mansoni*	see [9]	—	—	—
Aquaporins		*Caenorhabditis elegans*	NP_001024758.1<br>NP_001022480.1<br>NP_001021552.2<br>NP_508515.2<br>NP_505727.1<br>NP_505691.2<br>NP_505512.3<br>NP_502044.1<br>NP_499821.2<br>NP_496105.1<br>NP_495973.1<br>NP_495510.1	Ciccas_006023-T1		Aquaporin/Aquaporin-3
		S. mansoni	ACI31185.1			
				Ciccas_008834-T1		unknown
foraging (FOR)		*Drosophila melanogaster*	NP_001356955.1<br>NP_001356896.1	Ciccas_005236-T1	Group 1:<br>all	cGMP-dependent protein kinase

	NP_001356892.1<br>NP_001334731.1<br>NP_001162858.1<br>NP_001014464.1<br>NP_995629.1<br>NP_995628.1<br>NP_995626.1<br>NP_599146.1<br>NP_477490.1<br>NP_477489.1<br>NP_477487.1	Ciccas_ 006777-T1	Group 2:<br>all except *K. tanganicanus*	cGMP-dependent protein kinase
