# Supplementary material for: Adaptive evolution of stress response genes in parasites aligns with host niche diversity
Source: BMC Biol. 2025 Jan 13;23:10. doi: 10.1186/s12915-024-02091-w (PMC11727194; doi:10.1186/s12915-024-02091-w)
Supplement: Supplementary file 5 — Additional file 5. Stress gene tree topologies produced under the maximum likelihood criterion. Abbreviations: Ccas–Cichlidogyrus casuarinus, Ccir–C. cirratus, Chal–C. halli, Ckap–C. sp. ‘kapembwa’, Cscl–C. sclerosus, Cthu–C. thurstonae, Ctil–C. tilapiae, Czam–C. zambezensis, Slon–Scutogyrus longicornis, Klim–Kapentagyrus limnotrissae, Ktan–K. tanganicanus. [file 12915_2024_2091_MOESM5_ESM.pdf]

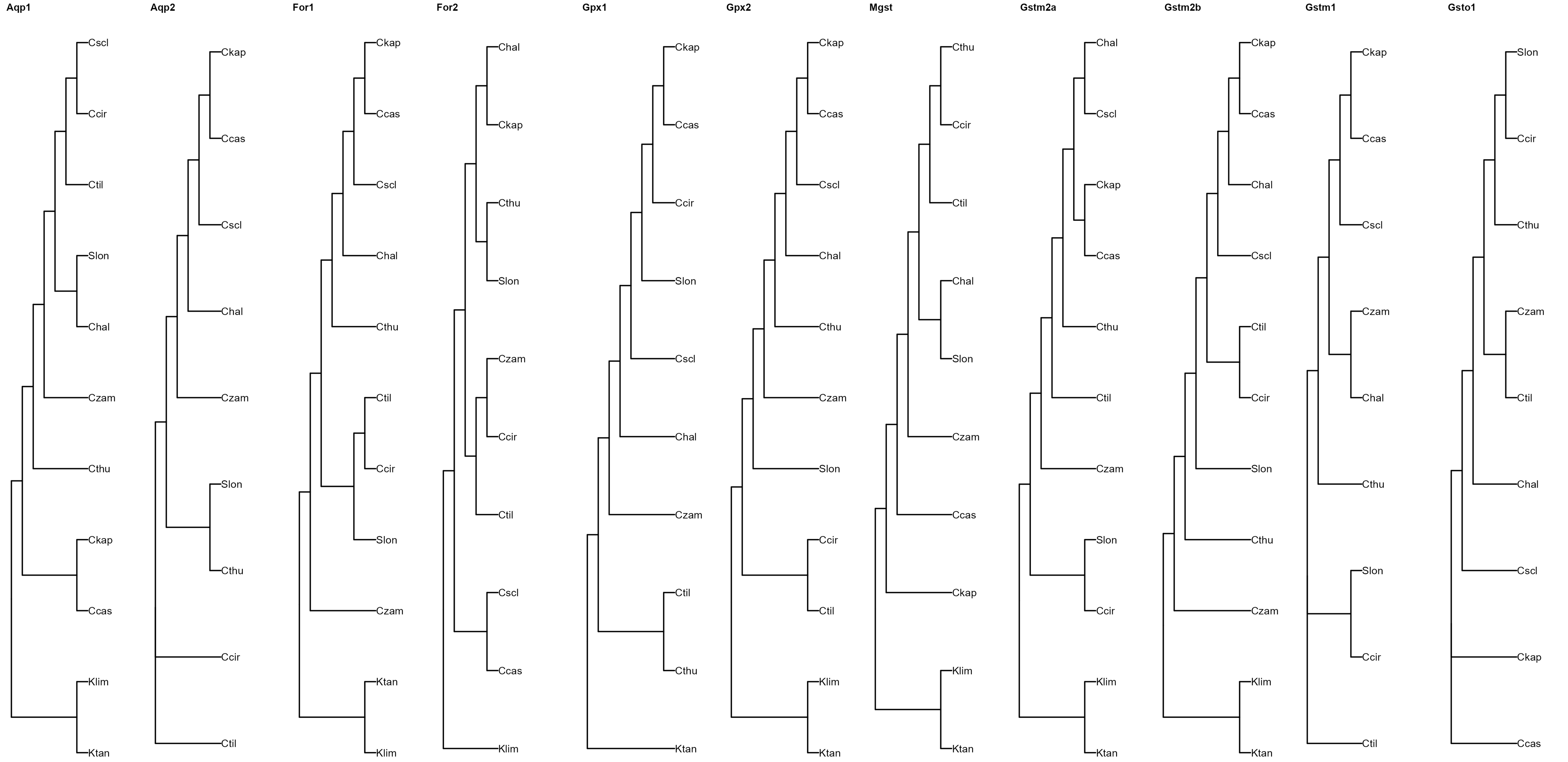
Aqp1
Cscl
Ccir
Ctil
Slon
Chal
Czam
Cthu
Ckap
Ccas
Klim
Ktan
Aqp2
Ckap
Ccas
Cscl
Chal
Czam
Slon
Cthu
Ccir
Ctil
For1
Ckap
Ccas
Cscl
Chal
Cthu
Ctil
Ccir
Slon
Czam
Ktan
Klim
For2
Chal
Ckap
Cthu
Slon
Czam
Ccir
Ctil
Cscl
Ccas
Klim
Gpx1
Ckap
Ccas
Ccir
Slon
Cscl
Chal
Czam
Ctil
Cthu
Ktan
Gpx2
Ckap
Ccas
Cscl
Chal
Cthu
Czam
Slon
Ccir
Ctil
Klim
Ktan
Mgst
Cthu
Ccir
Ctil
Chal
Slon
Czam
Ccas
Ckap
Klim
Ktan
Gstm2a
Chal
Cscl
Ckap
Ccas
Cthu
Ctil
Czam
Slon
Ccir
Klim
Ktan
Gstm2b
Ckap
Ccas
Chal
Cscl
Ctil
Ccir
Slon
Cthu
Czam
Klim
Ktan
Gstm1
Ckap
Ccas
Cscl
Czam
Chal
Cthu
Slon
Ccir
Ctil
Gsto1
Slon
Ccir
Cthu
Czam
Ctil
Chal
Cscl
Ckap
Ccas

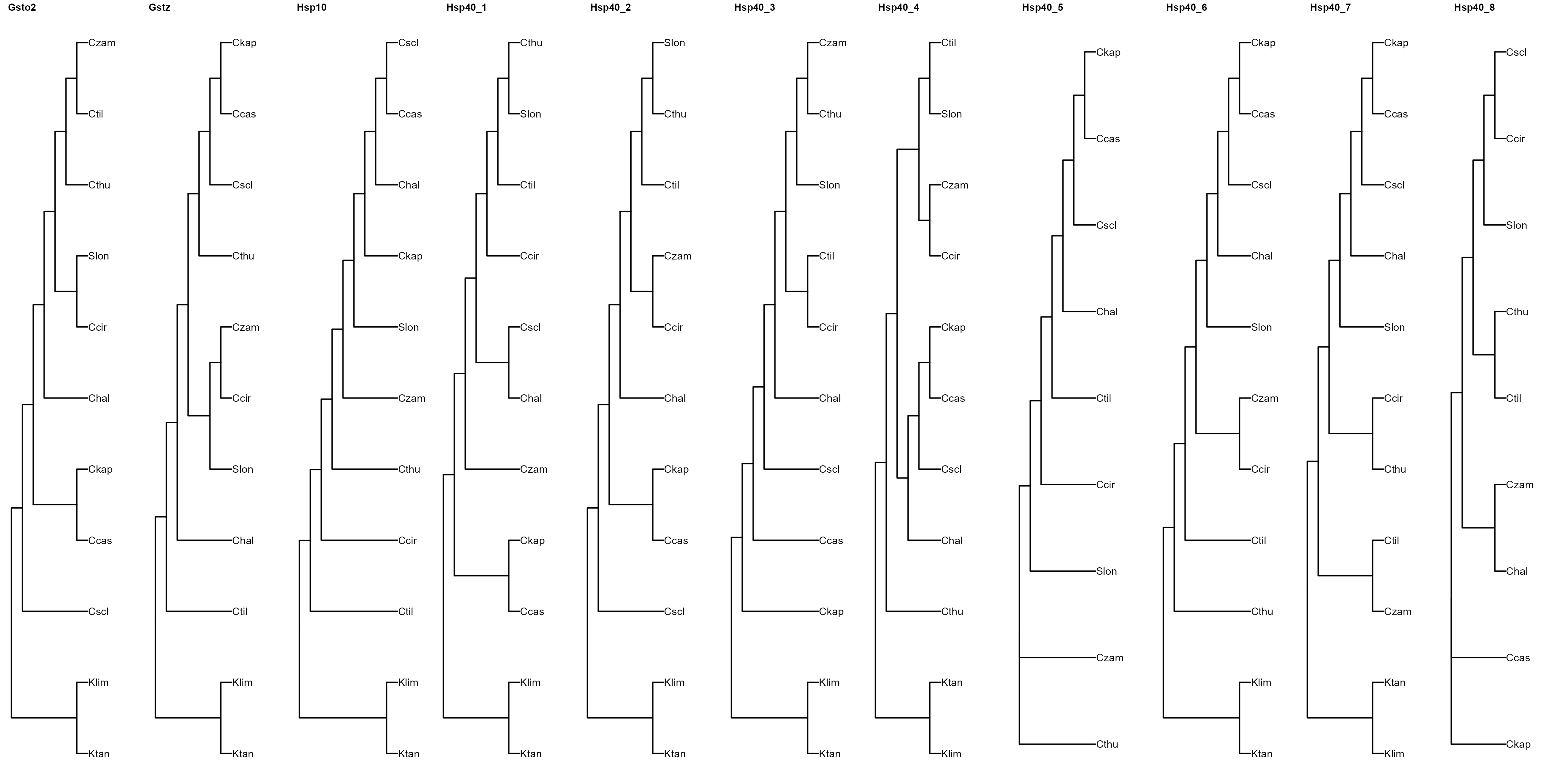
Gsto2
Czam
Ctil
Cthu
Slon
Ccir
Chal
Ckap
Ccas
Cscl
Klim
Ktan
Gstz
Ckap
Ccas
Cscl
Cthu
Czam
Ccir
Slon
Chal
Ctil
Klim
Ktan
Hsp10
Cscl
Ccas
Chal
Ckap
Slon
Czam
Cthu
Ccir
Ctil
Klim
Ktan
Hsp40_1
Cthu
Slon
Ctil
Ccir
Cscl
Chal
Czam
Ckap
Ccas
Klim
Ktan
Hsp40_2
Slon
Cthu
Ctil
Czam
Ccir
Chal
Ckap
Ccas
Cscl
Klim
Ktan
Hsp40_3
Czam
Cthu
Slon
Ctil
Ccir
Chal
Cscl
Ccas
Ckap
Klim
Ktan
Hsp40_4
Ctil
Slon
Czam
Ccir
Ckap
Ccas
Cscl
Chal
Cthu
Ktan
Klim
Hsp40_5
Ckap
Ccas
Cscl
Chal
Ctil
Ccir
Slon
Czam
Cthu
Hsp40_6
Ckap
Ccas
Cscl
Chal
Slon
Czam
Ccir
Ctil
Cthu
Klim
Ktan
Hsp40_7
Ckap
Ccas
Cscl
Chal
Slon
Ccir
Cthu
Ctil
Czam
Ktan
Klim
Hsp40_8
Cscl
Ccir
Slon
Cthu
Ctil
Czam
Chal
Ccas
Ckap

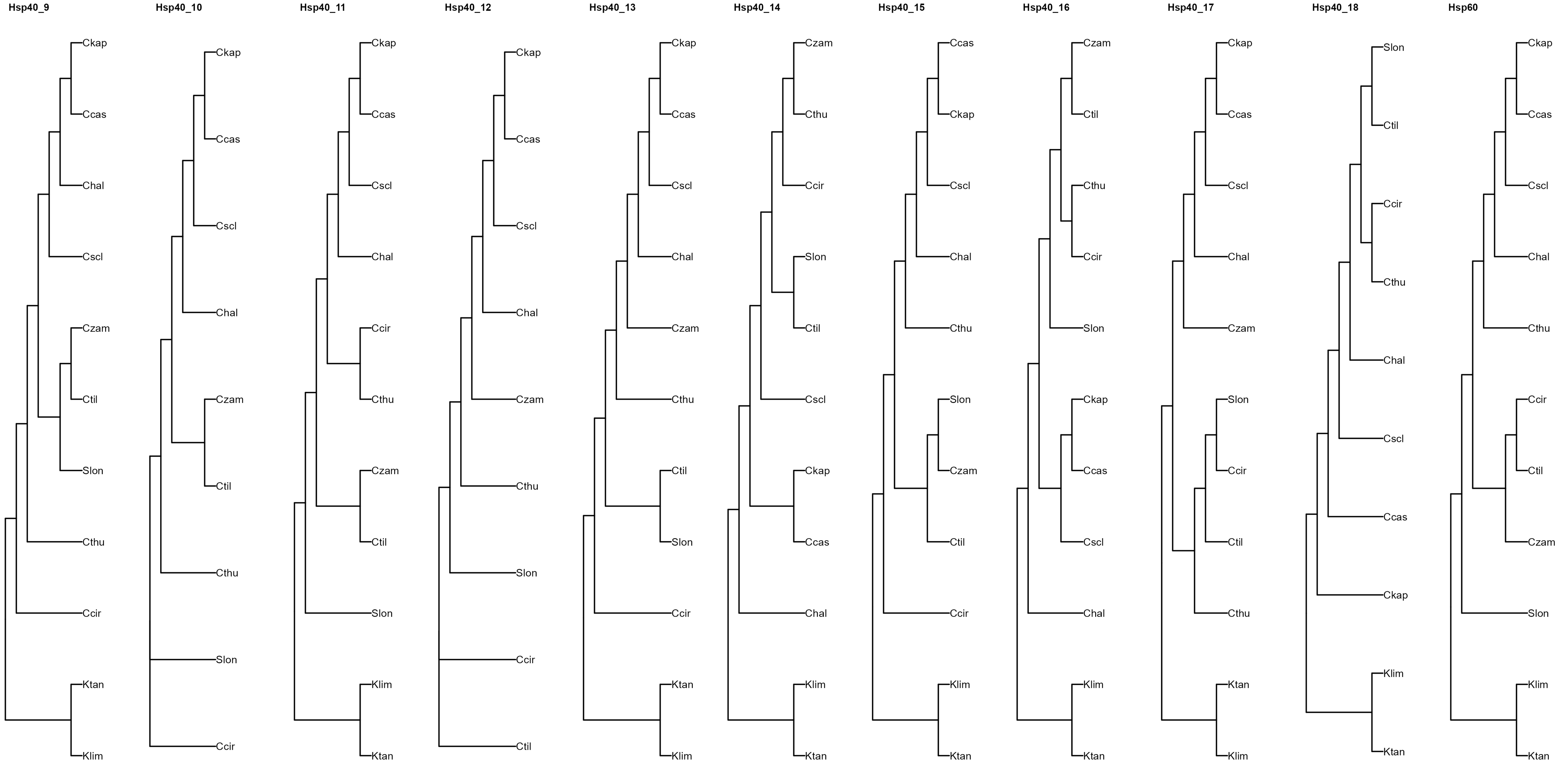
Hsp40_9
Ckap
Ccas
Chal
Cscl
Czam
Ctil
Slon
Cthu
Ccir
Ktan
Klim
Hsp40_10
Ckap
Ccas
Cscl
Chal
Czam
Ctil
Cthu
Slon
Ccir
Hsp40_11
Ckap
Ccas
Cscl
Chal
Ccir
Cthu
Czam
Ctil
Slon
Klim
Ktan
Hsp40_12
Ckap
Ccas
Cscl
Chal
Czam
Cthu
Slon
Ccir
Ctil
Hsp40_13
Ckap
Ccas
Cscl
Chal
Czam
Cthu
Ctil
Slon
Ccir
Ktan
Klim
Hsp40_14
Czam
Cthu
Ccir
Slon
Ctil
Cscl
Ckap
Ccas
Chal
Klim
Ktan
Hsp40_15
Ccas
Ckap
Cscl
Chal
Cthu
Slon
Czam
Ctil
Ccir
Klim
Ktan
Hsp40_16
Czam
Ctil
Cthu
Ccir
Slon
Ckap
Ccas
Cscl
Chal
Klim
Ktan
Hsp40_17
Ckap
Ccas
Cscl
Chal
Czam
Slon
Ccir
Ctil
Cthu
Ktan
Klim
Hsp40_18
Slon
Ctil
Ccir
Cthu
Chal
Cscl
Ccas
Ckap
Klim
Ktan
Hsp60
Ckap
Ccas
Cscl
Chal
Cthu
Ccir
Ctil
Czam
Slon
Klim
Ktan

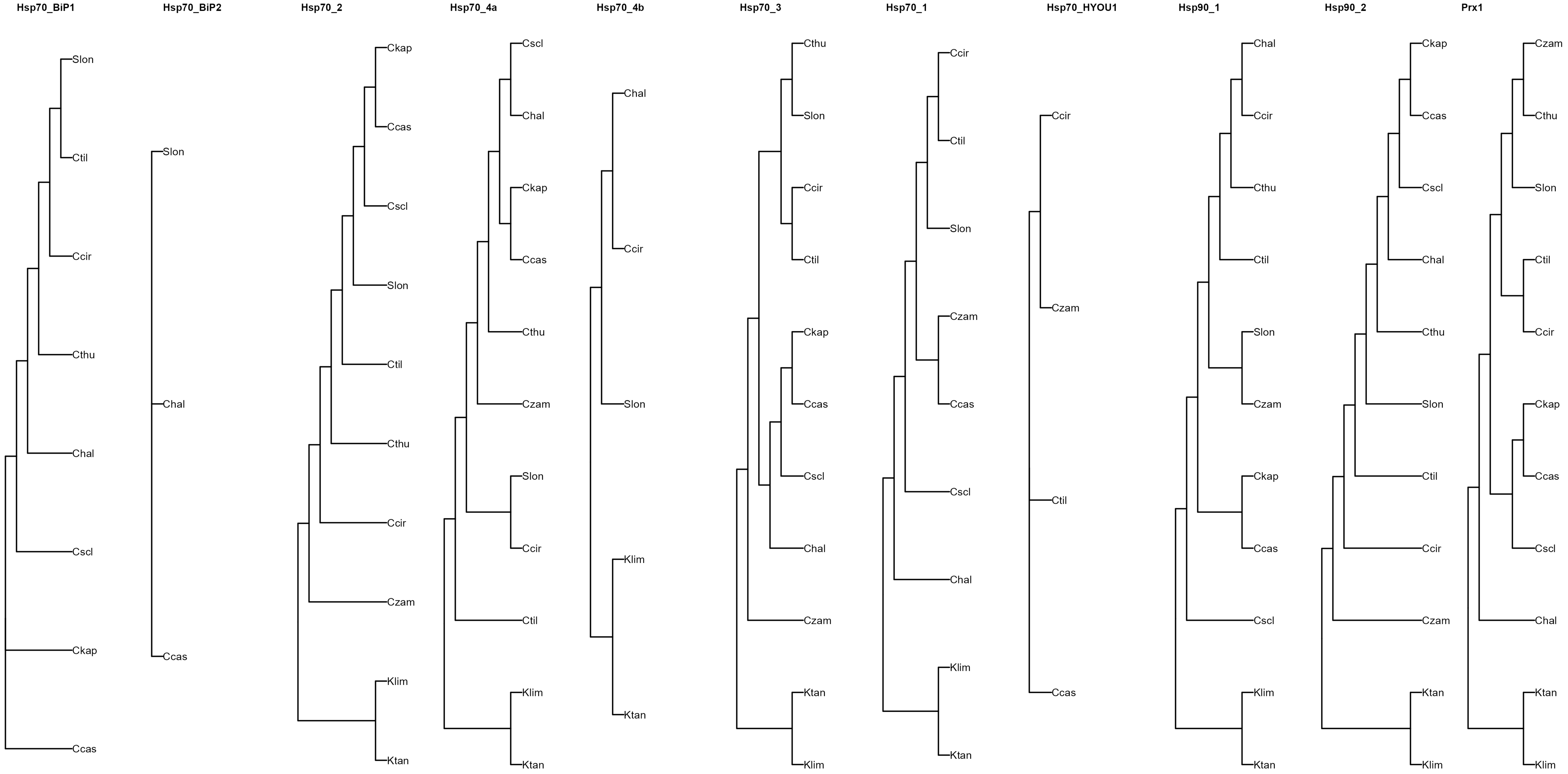
Hsp70_BiP1
Slon
Ctil
Ccir
Cthu
Chal
Cscl
Ckap
Ccas
Hsp70_BiP2
Slon
Chal
Ccas
Hsp70_2
Ckap
Ccas
Cscl
Slon
Ctil
Cthu
Ccir
Czam
Klim
Ktan
Hsp70_4a
Cscl
Chal
Ckap
Ccas
Cthu
Czam
Slon
Ccir
Ctil
Klim
Ktan
Hsp70_4b
Chal
Ccir
Slon
Klim
Ktan
Hsp70_3
Cthu
Slon
Ccir
Ctil
Ckap
Ccas
Cscl
Chal
Czam
Ktan
Klim
Hsp70_1
Ccir
Ctil
Slon
Czam
Ccas
Cscl
Chal
Klim
Ktan
Hsp70_HYOU1
Ccir
Czam
Ctil
Ccas
Hsp90_1
Chal
Ccir
Cthu
Ctil
Slon
Czam
Ckap
Ccas
Cscl
Klim
Ktan
Hsp90_2
Ckap
Ccas
Cscl
Chal
Cthu
Slon
Ctil
Ccir
Czam
Ktan
Klim
Prx1
Czam
Cthu
Slon
Ctil
Ccir
Ckap
Ccas
Cscl
Chal
Ktan
Klim
