# Supplementary material for: Adaptive evolution of stress response genes in parasites aligns with host niche diversity
Source: BMC Biol. 2025 Jan 13;23:10. doi: 10.1186/s12915-024-02091-w (PMC11727194; doi:10.1186/s12915-024-02091-w)
Supplement: Supplementary file 6 — Additional file 6. Sampling data of collected specimens including reference for sampling campaigns and published whole-genome sequencing data. [file 12915_2024_2091_MOESM6_ESM.pdf]

Additional File 6. Sampling data of collected specimens including reference for sampling campaigns and published whole-genome sequencing data.

Species	Host	Country	Locality	LAT	LON	Date	Pool size	Estimated coverage*	Sampling campaign	Genome sequences
Cichlidogyrus casuarinus Pariselle, Muterezi Bukinga & Vanhove, 2015	*Hemibates stenosoma* (Boulenger, 1901)	Zambia	Lake Tanganyika, Mpulungu	-08.7667	31.1167	09/2018 09/2019	80	825	[60]	This study
C. cirratus Paperna, 1964	*Oreochromis niloticus* (L., 1758)	Cameroon	Yaoundé, Ponds at Ecoparque	3.78582	11.4883	13/02/2022	50	643	[58]	This study
C. halli (Price & Kirk, 1967)	*O. niloticus*	Cameroon	Yaoundé, Fishing ponds of Obili, 'Projet de Promotion de l'Entreprenariat Aquacole (P.P.E.A.)	3.85672	11.4958	14/02/2020	50	1374	[58]	This study
C. sclerosus Paperna & Thurston, 1969	*O. niloticus*	Zimbabwe	Kariba, Lake Kariba at discharge channel of crocodile farm	-16.5495	28.8616	27/10/2019	50	365	[58]	This study
C. sp. 'kapembwa'[†,‡]	*Callochromis macrops* (Boulenger, 1898)	Zambia	Lake Tanganyika, Kalambo Lodge	-8.6539	31.1954	01/10/2019	52	1510	[59][§]	This study
C. thurstonae Ergens, 1981	*O. niloticus*	Cameroon	Mbalmayo, River Fala (affluent of the river So'o)	3,318972	11,48519	17/02/2020	50	556	[58]	This study
C. tilapiae Paperna, 1960	*O. niloticus*	Cameroon	Mbalmayo, Ponds Capfort	3.532733	11.51642	17/02/2020	50	482	[58]	This study
C. zambezensis Douëllou, 1993	*Serranochromis* cf. *thumbergi*	Dem. Rep. Congo	Province Haut-Katanga, Kiniama, Kafubu River, near Catholic Mission	-11.4777	28.3089	27/07/2019	1	768	[58]	This study
Scutogyrus longicornis Paperna & Thurston, 1969	*Oreochromis niloticus*	Cameroon	Édéa, small affluent stream (Mboue) of Sanaga River running through the village	3.79215	10.13303	11–12/02/2022	50	638	[58]	This study
Kapentagyrus limnotrissae (Paperna, 1973)	*Limnothrissa miodon* (Boulenger, 1906)	Zambia	Lake Tanganyika, Mpulungu	-08.7667	31.1167	08/2018	50	5451	[59]	[59]
K. tanganicanus Kmentová, Gelnar & Vanhove, 2018	*L. miodon*	Zambia	Lake Tanganyika, Mpulungu	-08.7667	31.1167	12/04/2018	85	8292	[59]	[59]

[*] Based on genome size of draft genome assembly for *C. casuarinus* (66.086 MB). [†] Species currently under description. [‡] Whole-genome pre-amplification was applied. [§] Sample location not mentioned in cited publication.
